# Advanced approach combines integrated weight water quality index and potential toxic elements for environmental and health risk assessment supported by simulation technique in Oued Souf, Algeria

**DOI:** 10.1038/s41598-024-68854-1

**Published:** 2024-08-01

**Authors:** Mohamed Hamdy Eid, Ahmed A. Tamma, Omar Saeed, András Székács, Mostafa R. Abukhadra, Ahmed M. El-Sherbeeny, Czímer Bence, Viktoria Mikita, Attila Kovács, Péter Szűcs

**Affiliations:** 1 Institute of Environmental Management, Faculty of Earth Science, University of Miskolc, Miskolc, 3515 Hungary; 2 Geology Department, Faculty of Science, Beni-Suef University, Beni-Suef, 65211 Egypt; 3 Institute of Environmental Engineering, Faculty of Environmentsl Engineering and Geodesy, Wroclaw University of Environmental and Life Sciences, 50-363 Wrocław, Poland; 4 Doctoral School of Environmental Science, Hungarian University of Agriculture and Life Sciences (MATE), Páter Károly u. 1, Gödöllő, 2100 Hungary; 5 Agro-Environmental Research Centre, Institute of Environmental Sciences, Hungarian University of Agriculture and Life Sciences, Páter Károly u. 1, Gödöllő, 2100 Hungary; 6 Industrial Engineering Department, College of Engineering, King Saud University, P.O. Box 800, 11421 Riyadh, Saudi Arabia

**Keywords:** PTEs, IWQI, Environmental and health risk, Monte Carlo simulation, Oued Souf, Geochemistry, Environmental sciences, Hydrology, Risk factors

## Abstract

The current research study evaluated the health and environmental risks issues associated with potentially toxic elements (PTEs) in the complex terminal aquifer located in the Algerian desert. The methods used included principal component and cluster (dendrogram) analysis to estimate source of ions and contamination. Various indices such as the Heavy Metal Pollution Index (HPI), Metal Index, hazard quotient, hazard index (HI), and cancer risk (CR) were applied to assess both environmental and human health risks. Furthermore, the Monte Carlo method was applied for probabilistic assessment of carcinogenic and non-carcinogenic risks through oral and dermal exposure routes in both adults and children. The results revealed that approximately 16% of the samples fell within the low pollution category (HPI < 100), indicating relatively lower levels of heavy metal contamination. However, the remaining 84% of the samples exhibited high pollution levels, indicating a significant presence of heavy metal pollutants in the northeastern part of the investigated area. The calculated average risk index (RI) for the collected samples was 18.99, with a range from 0.03 to 103.21. This indicates that a large portion, 82% of the samples, could cause low ecological risk (RI < 30), whereas the remaining 18% indicate a significant environmental pollution risk. The HI for oral ingestion showed that adults had HI values ranging from 0.231 to 1.54, while children exhibited higher values, ranging from 0.884 to 5.9 (Fig. 5a). For dermal exposure, HI values in adults ranged from 2.71E−07 to 8.74E−06 and in children, from 2.18E−06 to 7.03E−05. These findings highlight the potential non-carcinogenic risks associated with oral exposure to PTEs and underscore the increased vulnerability of children to metals such as Fe, Mn, Pb, and Cr. Most samples showed CR exceeding 1 × 10^−4^ for chromium (Cr) and lead (Pb), indicating a significant vulnerability to carcinogenic effects in both children and adults.

## Introduction

In developing nations, the swift pace of industrialization, economic growth, and urbanization is a major driver of increased environmental pollution, raising concerns both nationally and internationally^1–7^. Industries such as Petroleum-based chemicals and heavy automobiles manufacturing emit contaminants, including heavy metals, Organics, microplastics, pesticides, and newly developing pollutants, which threaten human health, groundwater resources, sustainable development, environmental agencies^1–19^. Recent global studies have found groundwater pollution with metals like lead, manganese, iron, cadmium, copper, and chromium^7,8,10,20^. Cadmium pollution, particularly prevalent in Asia and Africa, endangers food and water supplies due to its high toxicity and tendency to leach into soil and bioaccumulate in ecosystems, even at low concentrations^21^.

Groundwater pollution with heavy metals is associated with serious health issues, including degenerative neurological conditions, kidney harm, cardiovascular and respiratory diseases, and cancer^22,23^. Potential toxic elements (PTEs) are naturally persistent and develop in groundwater, making it a key exposure route for people^24^. As a result, public authorities regularly monitor PTE levels to reduce potential health risks. Given the vital importance of groundwater and the challenges it encounters, this study examines the various risks posed to humans and the environment by heavy metal contamination in the El-Oued region.

Although iron (Fe), and manganese (Mn) are essential for metabolic processes, they pose health hazards when their quantities in potable water surpass the allowable limits. Heavy metals can reach our bodies through ingestion, dermal contact, and inhalation^25–27^. These pollutants are prevalent in potable water sources, including groundwater and surface water^8,28–31^, as well as in vegetables, and air^32^. The environmental presence of these metals is primarily due to industrial processes and medical, domestic, agricultural, and technological events. When heavy metal concentrations in potable water surpass the thresholds set by worldwide organizations, it can result in numerous health issues^22^. Safeguarding environmental and human health requires comprehensive water quality assessments. This begins with measuring water quality and pinpointing pollution routes to reduce contamination amounts. Proven methodologies for assessing the ecological, environmental, and individual health concerns linked to PTEs (Fe, Cr, Mn, and Pb) include several indices (MI, HPI, HI, HQ, and CR), all of which can be enhanced through integration with Monte Carlo simulations for greater accuracy and reliability^23,32–40^. Moreover, the principal component (PC) analysis, along with the cluster analysis are critical methodologies for scaling the heavy metals routes and elucidating hydrochemical procedures in groundwater and surface water^8,9,13,31,41^. Globally, groundwater supplies are highly compromised by contamination and depletion, a challenge that also affects the Algerian deserts, where El-Oued significantly depends on groundwater for consumption and agricultural needs^42^. Reports show that more than one billion individuals cannot obtain adequate potable and agricultural water, causing around 25,000 fatalities annually in emerging nations^43^.

Semi-arid nations like Algeria confront significant water scarcity challenges, exacerbated by the looming threat of water resource pollution. Groundwater reservoirs in the northeastern Algerian desert serve as the second most crucial water source for irrigation, potable consumption, and industrial operations^44,45^.

The booming demands from population growth, agricultural expansion, and industrial development in this region have precipitated intensified extraction from deeper and shallower aquifers^44–46^. The aquifer system within the northeastern Algerian Sahara ranks among the biggest globally, characterized by a complex composition of continental intercalation and terminal aquifers^47^.

Groundwater abstraction in this region has witnessed a notable surge, escalating from 600 to 2120 Mm^3^/year between 1970 and 2020. The extraction of freshwater from the intricate final reservoir is mainly for drinking and agriculture, with total number of producing wells soaring to 203 bore holes by 2019^48–51^. The focal point of heightened abstraction lies within Debila and Oued cities. Given the gradual depletion of groundwater reserves and the deteriorating water quality within the studied areas, hydrochemical assessment and continuous water quality monitoring emerge as imperative measures for ensuring the sustainable and efficient control of the groundwater supply in this semi-confined aquifer^52,53^.

The present study aims to comprehensively investigate the ecological and individual health issues connected with PTEs across groundwater within the Oued-Souf aquifer (CT). The objectives of this research are as follows: To identify potential sources utilizing several statistical approaches, including PCA along with cluster and ionic ratio analysis and IDW interpolation; to elucidate the geochemical procedures governing the chemistry of water in the investigated area; to employ a novel technique that combines various water quality criteria and combined indicators (HPI, HQ, MI, CR, and HI) with Deterministic models or probability-based techniques, notably Monte Carlo approach, to measure both non-carcinogenic and carcinogenic health issues stemming from heavy metal contamination within the Oued-Souf aquifer; to leverage Python to streamline Monte Carlo simulations, thereby enhancing accuracy. This technique uses Python's rich libraries and analytical features to efficiently control uncertainty and unpredictability in input attributes, resulting in more reliable or accurate risk estimates. The combination of these approaches represents a substantial development in evaluating PTEs contamination within the Oued-Souf.

## Materials and methods

### Study area and geographic description

**Figure 1:**
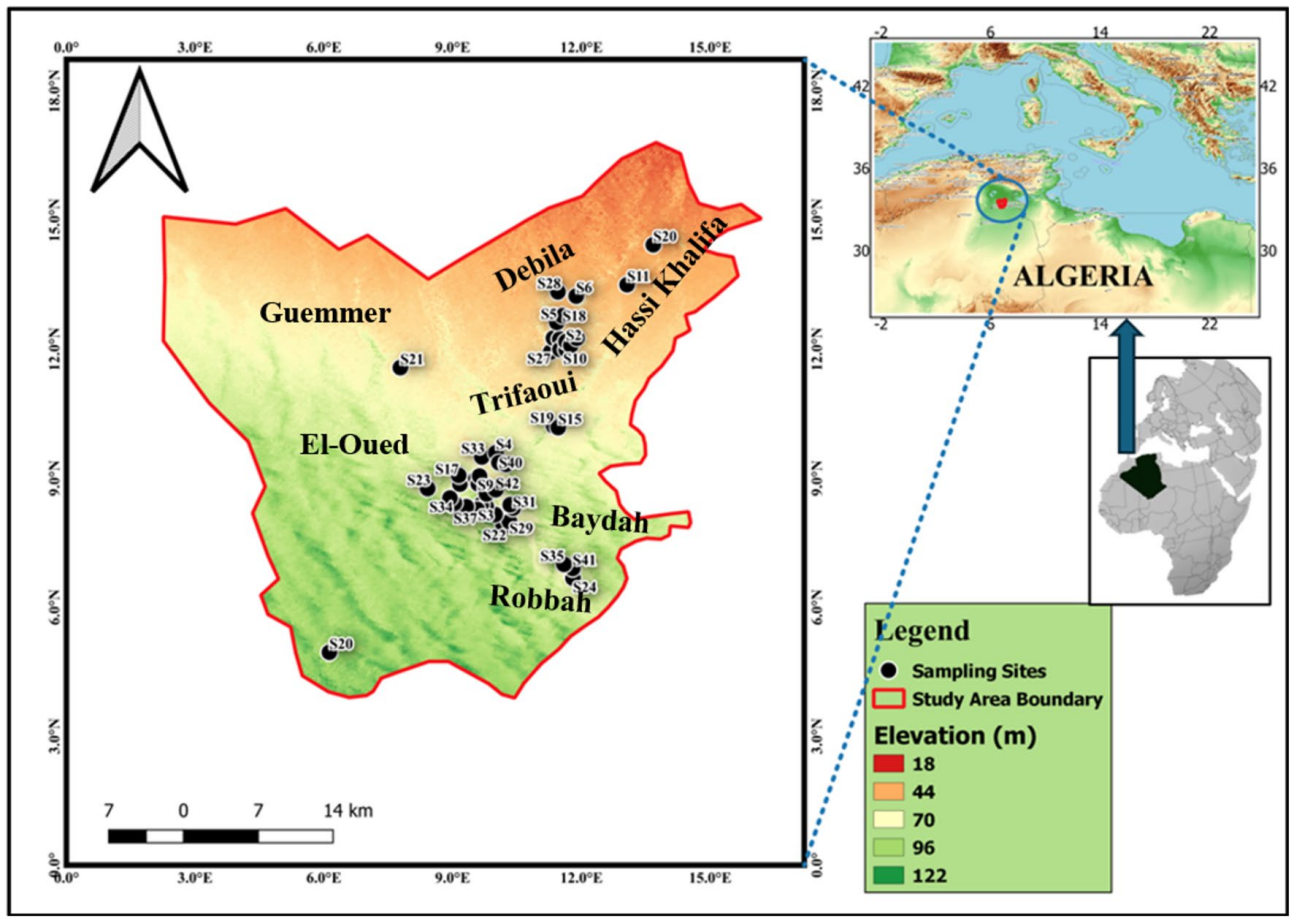
Location map of the study area and collected groundwater samples.

The research area is situated within the El-Oued and Debila regions in the northeastern expanse of the Algerian desert. Specifically, the Souf Valley occupies the geographic coordinates between latitude 33° 12′ 00″/33°35′ 00″ N and longitude 6° 40′ 00″/7°5′ 00″ E, encompassing an approximate area of 80,000 cubic meters. The population residing in this area is estimated to be around 900,000 individuals^44–46,54^. It is characterized by hot and arid climatic conditions; the region experiences an annual evapotranspiration rate of 1224 mm^55^. The investigated areas were strategically focused within the Debila and El-Oued sectors, with additional samples dispersed throughout the surrounding regions to assess spatial variations aligned with the course of groundwater discharge (Fig. 1). The sampling and location map was created using ArcGIS Pro 2.8.8 software.

**Figure 2:**
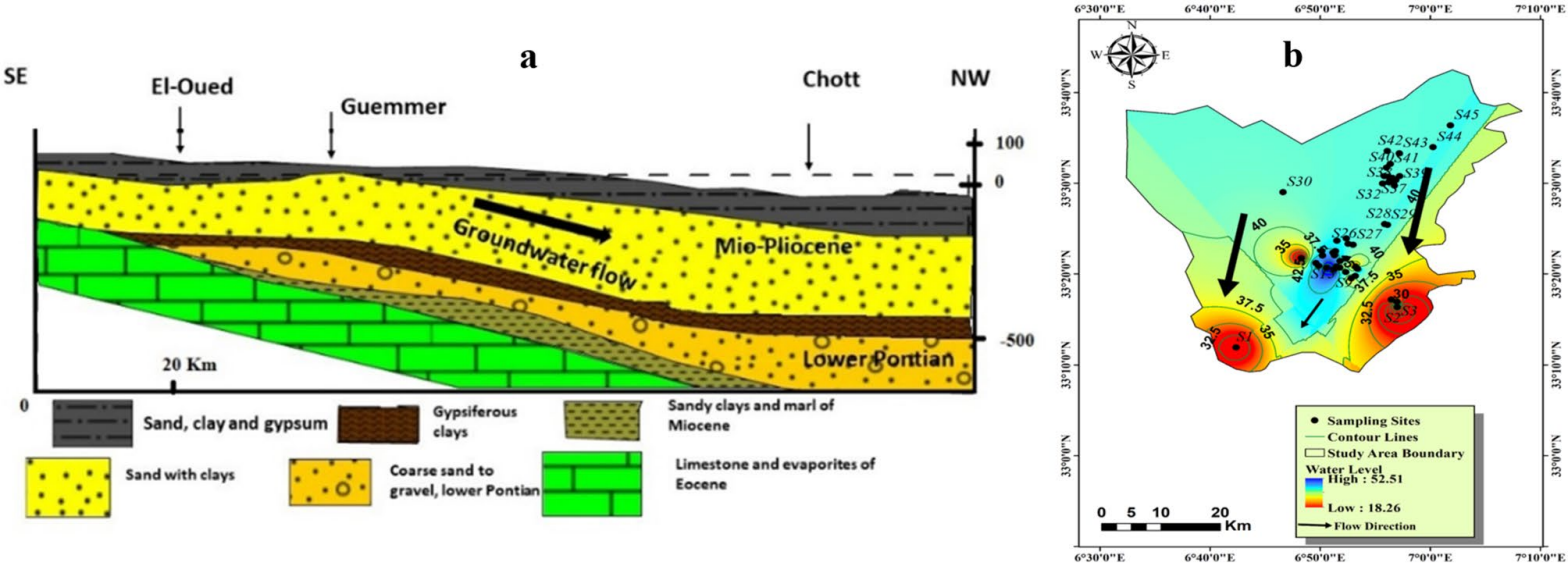
Geological cross-section (**a**) and groundwater flow direction (**b**) in the Oued-Souf (complex terminal) aquifer.

The hydrogeological framework of El-Oued encompasses three principal aquifers, arranged vertically as follows (Fig. 2a): the shallow quaternary aquifer, the complex terminal (CT) aquifer comprising formations from the lower Pontian and Mio-Pliocene periods, and the deep aquifer (Continental Intercalaire)^44–46,50,51,55^. The cross-section was edited after Eid^13^ and groundwater flow map was created using Surfer 16 software (Fig. 2a) and ArcGIS Pro 2.8.8 software (Fig. 2b).

The composition of the CT aquifer comprises dolomite and limestone formations at its lower section, followed by clastic deposits (gravel deposits) in the middle, and predominantly sand and sandstone formations (Mio-Pliocene) at its upper section. The CT aquifer exhibits a thickness of 300 m with a depth of 220 m. Groundwater within this aquifer migrates from the southwest (SW) to the northeast (NE), with the piezometric level being particularly high in Mih-Ouensa (SW) and gradually diminishing towards El-Oued (central part of the investigated study) and Trifaoui (NE)^55^. Extensive use of water resources for consumption and irrigation (Fig. 2b) has led to a decline in the piezometric head in specific areas (El-Oued and Trifaoui).

### Samples Collection and Analytical Approaches

In 2020, an overall of 45 water samples were methodically gathered from Forty-five groundwater production well locations (Fig. 1). To guarantee uniformity, samples were collected after 10 min of production, ensuring comprehensive representation from each investigated point. Before sampling, 500-ml polyethylene containers were extensively cleansed using chemicals, completely rinsed with filtered water, and submerged in a 10% HNO3 solution overnight. The obtained samples were then taken to a laboratory with a regulated temperature of 4 °C for further analysis. On-site pH measurements, temperature (°C), (TDS), (EC), were conducted using specialized instruments: pH meter and a digital thermometer (Hannah, Woonsocket, RI, USA). Additionally, TDS and EC were analyzed utilizing digital TDS and EC meters (HM digital, Redondo Beach, CA, USA). To ensure accuracy, all digital meters underwent standardization with deionized water and buffer solutions before the commencement of sample analysis. For cations analysis, the samples underwent filtration through 0.45 µm filters. Subsequently, ten drops of ultra-pure HNO3 were added to one set of samples. Calcium (Ca^2+^) and magnesium (Mg^2+^) contents were assessed using the EDTA titrimetric method, which employs ethylenediaminetetraacetic acid. Sodium (Na^+^) and potassium (K^+^) ion contents were measured utilizing a flame photometer (ELEX 6361, Eppendorf AG, Hamburg, Germany). Total hardness (TH) was evaluated using Eriochrome Black-T (C20H12N3O7SNa) and ammonium chloride (NH4Cl) indicators in an EDTA solution. To assess chloride (Cl^-^) concentrations, a titration method employing silver nitrate (AgNO_3_) and potassium chromate (K_2_CrO_4_) indicators was employed. For the detection of bicarbonate (HCO_3_^−^) and carbonate (CO_3_^2−^) concentrations, a titrimetric technique involving the solution of sulfuric acid (H2SO4) and methyl orange indicator was utilized. Additionally, Cl^-^ concentrations were determined through titration with silver nitrate. Concentrations of sulfate (SO_4_^2−^) and nitrate (NO_3_^−^) were tested using a spectrophotometer based on the visible ultraviolet (UV) spectrum (DR/2040- Loveland, CO, USA). Fe, Cr, Pb, and Mn were assessed through flame atomic absorption spectrometry (FAAS).

### Quality assurance and control

The water quality analysis followed the standard methodology specified by the American Public Health Association (APHA) in 2012. To ensure the accuracy of on-site testing equipment, we carefully standardized all instruments with deionized water and buffer solutions before starting sample analysis. Various quality assurance procedures were applied during the water sample examination. The analytical processes were validated by instrument calibration, accuracy, and predictability evaluations. Charging balance errors (CBE) were evaluated following field observations and then validated in the laboratory. The samples were examined in triplicate, and the average values were given as well. Equation (1) was used to analyze anion-cation balance errors based on the neutrality principal, which states that the sum of the number of cations equals the sum of the number of anions in meq/L. The CBE for all examined samples was within the permissible range of ± 5%.

1 $${\text{CBE }} = \frac{\Sigma Cations - \Sigma Anions}{{\Sigma Cations + \Sigma Anions}}*100$$

Furthermore, the quality assurance of the analytical procedure was double-checked through a meticulous examination involving Certified Reference Material (CRM) and the blank technique analysis.

### Indexing Techniques

#### Hydrochemical Evaluation method

In this investigation, the chloralkaline indices (CAI-I and CAI-II) (Eqs. 2–3) were employed to ascertain the mineral composition within the aquifers and the ionic exchanges occurring in groundwater systems.

2 $$CAI-I=\frac{{Cl}^{-}-({Na}^{+}-{Ca}^{+})}{{Cl}^{-}}$$

3 $$CAI-II=\frac{{Cl}^{-}-({Na}^{+}-{Ca}^{+})}{{So}_{4}^{2-}+{HCO}_{3}^{-}+{CO}_{3}^{2-}+{NO}_{3}^{2-}}$$

The hydrochemical evaluation methods included, Gibbs, CAI-I, CAI-II, piper, and Ionic ratios (Mg^2+^/Na^+^ vs Ca^2+^/Na^+^, HCO_3_^-^/Na^+^ vs Ca^2+^/Na^+^) plots to determine the water type of different water resources and the mechanism controlling the water chemistry. Digrammes software and Excel sheet were used for visualization of the graphs. The application of geochemical modeling based on the physicochemical parameters and heavy metals were utilized to determine the mineral saturation state and the main minerals have the most contribution for enrichment of elements in water through the water–rock interaction. The model extracted from PHREEQC showed the output as saturation index (SI) for each mineral with positive value indicating super saturation state of specific mineral and negative value for under saturation state while zero value indicates equilibrium state where the water is not able to dissolve or precipitate certain minerals. The saturation index was calculated based on standard equation^56,57^ using log ion activity product divided by solubility product (Eq. 4).

4 $$({\text{SI}} = {\mathrm{log }}\left( {{\text{IAP}}/{\mathrm{K}}} \right)$$

IAP stands for "ion activity product," and ${K}_{sp}$ stands for "solubility product" at a given temperature.

### Integrated weight water quality index (IWQI)

The Integrated Weight Water Quality Index (IWQI) is a ranking system that indicates the overall impact of physicochemical characteristics on water quality, as determined by the Water Quality Index (WQI). In this work, IWQI was used to evaluate water quality^58–61^. IWQI computation consists of five steps: entropy weighting, CRITIC-based weighting, determining integrated weights, calculating the integrated-weight water quality index, and evaluating groundwater quality using WQI values.

### Entropy weight calculation

The entropy-weighted water quality index (EWQI) is an approach to estimate water quality that relies on certain hydrochemical variables to calculate an overall entropy value^62,63^. The EWQI calculation technique consists of three phases, which are detailed below:

**In Step 1**, we estimated the eigenvalues of the matrix X using Eq. (5), where m and n represent the total number of investigated samples and hydrochemical parameters to be evaluated, respectively.

5 $${\text{x}}=\left[\begin{array}{ccc}{\text{x}}_{11}& {\text{x}}_{12}& {\text{x}}_{1\text{n}}\\ {\text{x}}_{21}& {\text{x}}_{22}& {\text{x}}_{2{\text{n}}}\\ {\text{x}}_{{\mathrm{m}}_{1}}& {\text{x}}_{{\mathrm{m}}_{2}}& {\text{x}}_{\mathrm{mn}}\end{array}\right]$$

**In Step 2**, we apply Eqs. (6) and (7) to calculate the standard matrix Y. Because of large dimensional differences in hydrochemical indicators, data normalization is required before computing the EWQI. Here, (*Xij*)*max* denotes the greatest value, while (*Xij*)*min* symbolizes the minimum value for the corresponding hydrochemical parameters.

6 $$Yij=\frac{Xij-\left(Xij\right)min}{(Xij)max-\left(Xij\right)min}$$

7 $${\text{Y}}=\left[\begin{array}{ccc}{\text{Y}}_{11}& {\text{Y}}_{12}& {\text{Y}}_{1\text{n}}\\ {\text{Y}}_{21}& {\text{Y}}_{22}& {\text{Y}}_{2\text{n}}\\ {\text{Y}}_{{\text{m}}_{1}}& {\text{Y}}_{{\text{m}}_{2}}& {\text{Y}}_{\text{mn}}\end{array}\right]$$

In Step 3, we implement Eqs. (8–10) to calculate information entropy ej and entropy weight wj. Here, Pij represents the index j value of sample I.

8 $$ej=\frac{1}{Lnm}{\sum }_{i=1}^{m}\left({\text{Pij}}\times{ \text{LnPij}}\right)$$

9 $$Pij=\frac{Yij}{{\sum }_{i}^{m}\left(\text{Yj}\right)}$$

10 $$Wj1=\frac{1-ej}{{\sum }_{i=1}^{n}\left(1-ej\right)}$$

### Objective weight (CRITIC Method)

In this study, the Criteria Importance Through Inter-criteria Correlation (CRITIC) technique was employed to compute the objective weights of variables and overcome the constraints of conventional information entropy methods. The objective weight can be determined using the following equations (Eqs. 11–13):

11 $${r}_{ij}=\frac{\sum \left({x}_{ij}-\overline{{x }_{ij}}\right)\left({y}_{ij}-\overline{{y }_{ij}}\right)}{\sqrt{\sum {\left({x}_{ij}-\overline{{x }_{ij}}\right)}^{2}\Sigma {\left({y}_{ij}-\overline{{y }_{ij}}\right)}^{2}}}$$

12 $$Sj=\updelta j{\sum }_{i=1}^{m}\left(1-{\text{rij}}\right)$$

13 $$Wj2=\frac{Sj}{{\sum }_{j=1}^{m}\left(Sj\right)}$$

In this context, wj2 represents the objective weight of the jth parameter, with m denoting the total number of variables. Sj represents the quantity of information, while δj represents the standard deviation of the jth parameter.

### Integrated-weight estimation:

The following equations (Eqs. 14–16) were utilized to determine the integrated-weight Wj:

14 $${\text{W}}_{j} = pwj{1}\, + \,({1}\, - \,p)wj{2}$$

15 $$p={\sum }_{j=1}^{m}\left[{\left({W}_{j}-{w}_{j1}\right)}^{2}{\left({W}_{j}-{w}_{j}2\right)}^{2}\right]$$

16 $$Wj=\frac{wj1\times wj2}{{\sum }_{j=1}^{m}\left(wj1\times wj2\right)}$$

In this section, p represents a preference coefficient, with p$\epsilon$ [0,1].

### Integrated Weight Water Quality Index (IWQI)

After estimating the entropy weight, wj1, and the objective weight, wj2, the following formulas (Eqs. 17, 18) are used for calculating the Integrated Weight Water Quality Index (IWQI):

17 $$Qj = \frac{Cj - Cjp}{{Sj - Cjp}} \times {1}00$$

18 $$IWQI={\sum }_{j=1}^{m}\left(WjQj\right)$$

In the equations, j corresponds to the experimental concentration of every parameter in mg per liter, and Cjp reflects the variable's standard value in pure water used for drinking. It is zero for all variables except the pH level, which has a standard score of seven. The standard value (Sj) for each physicochemical factor determined according to WHO standards^64^ is reported in mg/L. Table 1 represent the input parameters and Integrated Weight.

**Table 1:** The parameters used in calculation of integrated weight water quality index (IWQI).

parameter	Unit	Cjp (variable's standard value in pure water)	Sj (WHO 2011)	Wj (integrated weight)
TDS	mg/L	0	1000	0.045074
pH		7	7.5	0.045624
EC	µS/cm	0	1500	0.043698
Na	mg/L	0	400	0.047656
K	mg/L	0	12	0.070666
Mg	mg/L	0	150	0.046403
Ca	mg/L	0	200	0.047419
Mn	mg/L	0	0.1	0.250271
Fe	mg/L	0	0.3	0.100279
Cl	mg/L	0	600	0.050539
SO_4_	mg/L	0	400	0.047423
HCO_3_	mg/L	0	200	0.054388
NO_3_	mg/L	0	45	0.11145
TH	mg/L	0	500	0.03911
				∑Wi = 1

According to IWQI values, water can be categorized into five distinct classes^58,61^. When the IWQI value is below 100 (excellent to good category), the water is deemed suitable for oral consumption and other uses. Between 100 and 150 indicates medium or intermediate quality, while the poor and extremely poor water quality falls between 150 and greater than 200.

### Heavy metal pollution index (HPI) and Heavy Metal Index (HMI)

The Pollution Index (HPI) is a useful measure to estimate the level of heavy metal pollution that affects water bodies^65^. The HPI is particularly useful in identifying the appropriateness of water for ingestion where heavy metals are present. It is calculated using attribute ratings and weighted mean calculations. Each pollutant characteristic is weighted, and a grading system ranging from 0 to 1 highlights the importance of each quality aspect or its relation to specified reference standards^7^. Equations (19 and 20) provide the particular computations used to determine the HPI.

19 $${\text{HPI}}=\frac{{\sum }_{{\text{i}}=1}^{\mathrm{n}}{\text{W}}_{\text{i}}{\mathrm{Q}}_{\text{i}}}{\sum_{\text{i}=1}^{\mathrm{n}}{\text{Wi}}}$$

In the formula, Qi is the sub-index factor, n represents the number of analyzed variables, wi is the weight assigned to each factor, calculated as 1/Si, where Si is the standard value for each variable. Qi is also the sub-index of the boundary, as defined by Eq. (20).

20 $${Q}_{i}={\sum }_{i=1}^{n}100 \times \frac{{C}_{i}}{{S}_{i}}$$

The HPI indicator calculates the levels (concentrations) of iron (Fe) and manganese (Mn). Heavy metal index is often assessed using a modified five-category scale. The categories are: Excellent water quality (HPI below 15); Good to intermediate quality (15 < HPI < 30); Poor to unsuitable quality (HPI > 30); Very poor quality (76 < HPI < 100); unsuitable (HPI > 100)^66,67^.

The Metal Index (MI) for potable water is one of the metrics that assess the combined impacts of PTEs on individual health, therefore determining potable water's overall purity or quality. This index is based on the principle that there is a proportional relationship between the level of PTEs and their toxicity^7^. Exposure to these metals can cause a variety of toxicological risks, both immediate and long-term, on different human organs^7,10,20^. Calculating the MI requires a comprehensive evaluation of current conditions. If the concentration exceeds its specified Higher Allowable Limits (HAL), it indicates a deterioration of groundwater quality. The MI concept was introduced by Tamasai and Cini and is defined by Eq. (21).

21 $$HMI=\sum_{i=1}^{i}\frac{{C}_{i}}{{HAL}_{i}}$$

where C_*i*_ represents the concentration of each investigated HM, whereas (HAL_*i*_) refers to the higher allowable limits for each element (*i*)th.

According to Withanachchi^68^, the Heavy Metal Index (HMI) is divided into six scales: There are five categories of contamination: very clean (HMI < 0.3), clean (0.3 < HMI < 1), somewhat polluted (1 < HMI < 2), moderately polluted (2 < HMI < 4), highly polluted (4 < HMI < 6), and seriously polluted (HMI > 6). These levels represent major health hazards. This grading method helps evaluate water resources quality in terms of PTE pollution and its possible influence or risk on individuals' health.

### Ecological risk index

The presented ecological risk indicator (RI) of hazardous metals, initially proposed by Hakanson^69^, is an approach for determining the risk connected with an abundance of HM in an ecosystem. As Xie point out, this score takes into account characteristics such as heavy metal concentrations, kinds, sensitivity, toxicity, and background levels^70^. While it can be applied to various scientific domains, it was especially used in this study to assess the ecological dangers associated with heavy metals in groundwater. The formula is given as follows (Eq. 22):

22 $$RI=\sum {E}_{r}^{i}={T}_{r}^{i}\times \left\{\frac{{c}^{i}}{c{i}_{bg}}\right\}$$

In the equation, ${E}_{r}^{i}$ reflects a substance's possible ecological indicator portion; ${T}_{r}^{i}$ depicts the specified metal's toxic reaction variable (Table S1); ${c}^{i}$ designates a typical level of PTEs in each sample, and $c{i}_{bg}$ represents the background score or value of every metal (Table S1). The RI represents the whole ecological impact. The risk indicator (RI) is classified into four categories or levels of possible risk: low, moderate, significant, and very high, with RI values < 30, 30 to 60, 60 to 120, and > 120, respectively^71^.

### Multivariate Statistical methods

Researchers frequently employ multivariate statistical methods to thoroughly understand groundwater condition and their fundamental chemistry. Our study combined trace metals, physical, and chemical attributes to investigate the intricate interactions among various factors and components within aquatic ecosystems. This framework used Principal Component Analysis (PCA) and Hierarchical Cluster Analysis (HCA) as analytical tools.

### Exposure to PTEs assessment

Drinking water contaminated with toxic metals poses risks of both non-carcinogenic and carcinogenic ailments in humans^67,72^. This study followed procedures established by the U.S. Environmental Protection Agency (USEPA) to measure the non-carcinogenic hazards connected with Cr, Fe, Mn, and Pb^20,73^. The USEPA's framework for health risk assessment, established in 2004, aims to evaluate the non-cancerous health risks posed by heavy metal factors in surface water and groundwater through ingestion, inhalation, and dermal contact. The primary hazard stems from directly ingested water and uptake via the skin^20,67,73,74^. This approach estimates the effect of PTEs ingested by humans using the CDI method as described in Eqs. (23 and 24), respectively^20,75,76^.

23 $${\text{CDI}}_{\text{oral}}=\frac{{\text{C}}_{\text{HMs}}\times {\text{EF}}\times {\text{IR}}}{\mathrm{AT}\times {\text{BW}}} \times \text{ED}$$

24 $${\text{CDI}}_{\text{dermal}}=\frac{{\text{C}}_{\text{HMs}}\times {\text{ET}}\times {\text{EF}}\times \text{Kp}\times \text{SA}\times \text{CF}}{\mathrm{BW}\times \text{AT}} \times \text{ED}$$

CDI refers to chronic daily intake (mg/kg/day), while *C*_HMs_ represent the concentration of each heavy metal (mg/L). The (IR) is the intake rate (children: 1.8 L/day; adults: 2.2 L/day). The (ED) indicates the Exposure duration (children: 6 years; adults: 70 years) with an exposure frequency (EF) of 350 days per year for both adults and children. K_*p*_ denotes the permeability coefficient (cm/h), as indicated in Table S1; ET signifies exposure time (0.58 h/day for adults and 1 h/day for children). SA denotes the skin area (18,000 cm^2^ for adults and 6600 cm^2^ for children). CF represents the unit conversion factor (1 × 10^–3^ L cm^-3^). BW stands for body weight (children: 15 kg; adults: 70 kg). AT refers to the average duration of carcinogenic hazards^20,73,77^.

### Non-carcinogenic risk assessment

This study evaluated the health hazards associated with Fe, Cr, Mn, and Pb (PTEs) in groundwater through risk assessment model created by USEPA. The non-carcinogenic risk index (HI) is a predictive model developed by the USEPA for assessing the health risks of chemical element combination^78^. The HI comprises two components: chronic daily ingestion (CDI) and the hazard quotient (HQ), represented by the following equations:

25 $${HQ}_{dermal/oral}=\frac{{\text{CDI}}_{\text{dermal}}{/\mathrm{CDI}}_{\text{oral}}}{{\mathrm{RfD}}_{\text{dermal}}{/\mathrm{RfD}}_{\text{oral}}}$$

26 $${RfD}_{dermal}={RfD}_{oral}\times ABS$$

27 $$HI={HQ}_{oral}+{HQ}_{dermal}$$

RfD (mg/kg/day) stands for the reference dose of a particular heavy metal. The RfD values for various heavy metals are provided in Table S1.

### Carcinogenic human health risk method

The carcinogenic risk (CR) can be determined by multiplying the CDI with the cancer slope factor (CSF), measured in per mg/kg/day (Eq. 28).

28 $$CR=CDI \times CSF$$

The tolerable range for such hazards is 1 × 10^−6^ to 1 × 10^−4^^20,79^. where CSF values are shown in (Table S1).

### Monte Carlo Simulation Techniques

In this investigation, Monte Carlo was utilized as a simulation technique to estimate the probability distributions of various attributes such as PTEs levels, exposure time and frequency, ingestion rates, absorption coefficients, skin surface area, and individual body weight. This approach or method aims to characterize the probability distributions and uncertainty reduction^7,10,20,67^. This technique allows for the prediction of the hazard quotient (HQ) and cancer risk (CR) for both oral and dermal exposure in two age groups (children and adults). By integrating this simulation with the USEPA's health risk assessment framework, we can assess the adverse effects of heavy metal exposure, estimating CR probability distributions. The analysis includes variables such as heavy metal concentrations and other related factors, as detailed in Eqs. (25–28). To ensure the reliability of the simulation, 10,000 iterations were performed using Python code (PyCharm Community Edition 2023.1.1). The consistency between actual and simulated HQ values calibrates the model's efficiency. The distribution approach for PTEs levels was based on 2020 data, while variables like ingestion rate, exposure duration, skin surface area, and body weight were modeled using normal distributions to accurately represent their real-world distributions.

## Results and discussions

### Measured parameters compared with standard limits

Physicochemical characteristics of the groundwater resources in deep aquifers (CT) play a significant role in the initial evaluation of its quality and suitability for agricultural and drinking use, serving as a useful method for identifying specific environmental issues, defining patterns, and disseminating information on water resources, geochemical processes, and water quality. The following parameters were utilized to categorize the suitability of groundwater for irrigation and drinking purposes in the selected deep aquifers. Meanwhile, the selected parameters like T^O^, pH, EC, TDS, K^+^, Na^+^, Mg^2+^, Ca^2+^, Cl^−^, SO_4_^2−^, HCO_3_^−^, CO_3_^2−^, and NO_3_^−^, and heavy metals (HMs) such as Fe, Cr, Pb, and Mn are altering the quality and productivity of the soil and could cause several environmental and health risks. The statistical methods of the analyzes parameters of 45 water samples were presented in Table 2.

**Table 2:** Statistical analysis of the different analyzed attributes in CT aquifers across the investigated area.

Parameter	Min	Max	Average	Parameter	Min	Max	Average
Temp	13.9	38.8	26.66383	K	12	42	33.30
pH	7	7.9	7.45	Cl	560	1127	822.25
Turbidity	0.074	11.5	1.71	SO_4_	532	840	697.53
TH	830	1350	1097.45	HCO_3_	106	195	135.50
EC	2640	4360	3646	NO_3_	0.83	31.54	15.82
TDS	1702	2790	2343	Fe	0.001	3.61	0.713
Ca	168	341	259	Mn	0.003	0.631	0.265
Mg	24.31	157.98	110	Cr	0.001	0.031	0.012
Na	210	540	364	Pb	0.001	0.039	0.019

The analyzed parameters were stated in milligram per liter excluding physical parameters; temperature (T^O^C), pH, and EC (µs/cm). The findings indicated a reduction in salinity in the path of groundwater (GW) flow. According to the statistical analysis, the minimum (min) and maximum (max) value was considered to evaluate the water condition or quality according to two well-known standards^64,80^. The salinity (TDS) ranged from 1702 to 2790 mg/L with average value of 2343 mg/L, which exceeds the permissible limits of drinking (1000 mg/L), while for irrigation 77% of water samples located in eastern and south part of the investigated area exceed the limits based on FAO standards (2000 mg/L). The water samples fell between neutral and slightly alkaline condition with pH values ranging from 7 to 7. 9. The concentration of calcium in all of the water samples fits the standards of irrigation water (FAO 1994) and fell between 168 and 340 mg/L, while for drinking purposes 86% of samples had value greater than permissible limit (> 200). The Debila area showed the uppermost value for Ca^2+^. Only 4.5% of samples showed a low concentration of Mg^2+^ within the permissible standard of the irrigation water, while the concentration of Mg was within the acceptable limits (below 150 mg/L) of drinking in the majority of samples. For Na^+^, the concentration was between minimum value of 210 mg/l and maximum value of 540 mg/l which fell in safe category for irrigation but not suitable for drinking in 17% of samples. The water samples in the western part of the study area are more enriched in sodium. The main dominant anions in the groundwater of Souf valley are SO_4_^2−^ and Cl^−^ with concentration range value of 532 to 840 mg/L and 560 to 1127 mg/l. The sulfates and chloride ions concentration did not exceed the limits of irrigation, while these ions levels exceeded the limits of drinking in the majority of samples. The obtained concentration of HCO_3_^-^ in all water samples showed an acceptable level for drinking and irrigation with a maximum value of 135.5 mg/l. Chemical pollution is linked to the availability of nitrates in groundwater. The potential toxic elements (PTEs) in the investigated study showed a wide variation in concentration ranging from 0.001 to 3.6, 0.003 to 0.6, 0.001 to 0.031, and 0.001 to 0.031 mg/l for Fe, Mn, Cr, and Pb respectively. The concentration range of PTEs in Oued Souf showed that 33.3%, 82%, 0%, 62.2% of samples were above the drinking permissible limit for Fe, Mn, Cr, and Pb respectively which could have environmental and health risk concern and it will be discuss in the current research study. Figure S1 demonstrates the distribution maps of all measured parameters to detect the most location could be affected by water quality deterioration. The distribution maps were created using ArcGIS Pro 2.8.8 software.

### Hydrochemical characteristics

**Figure 3:**
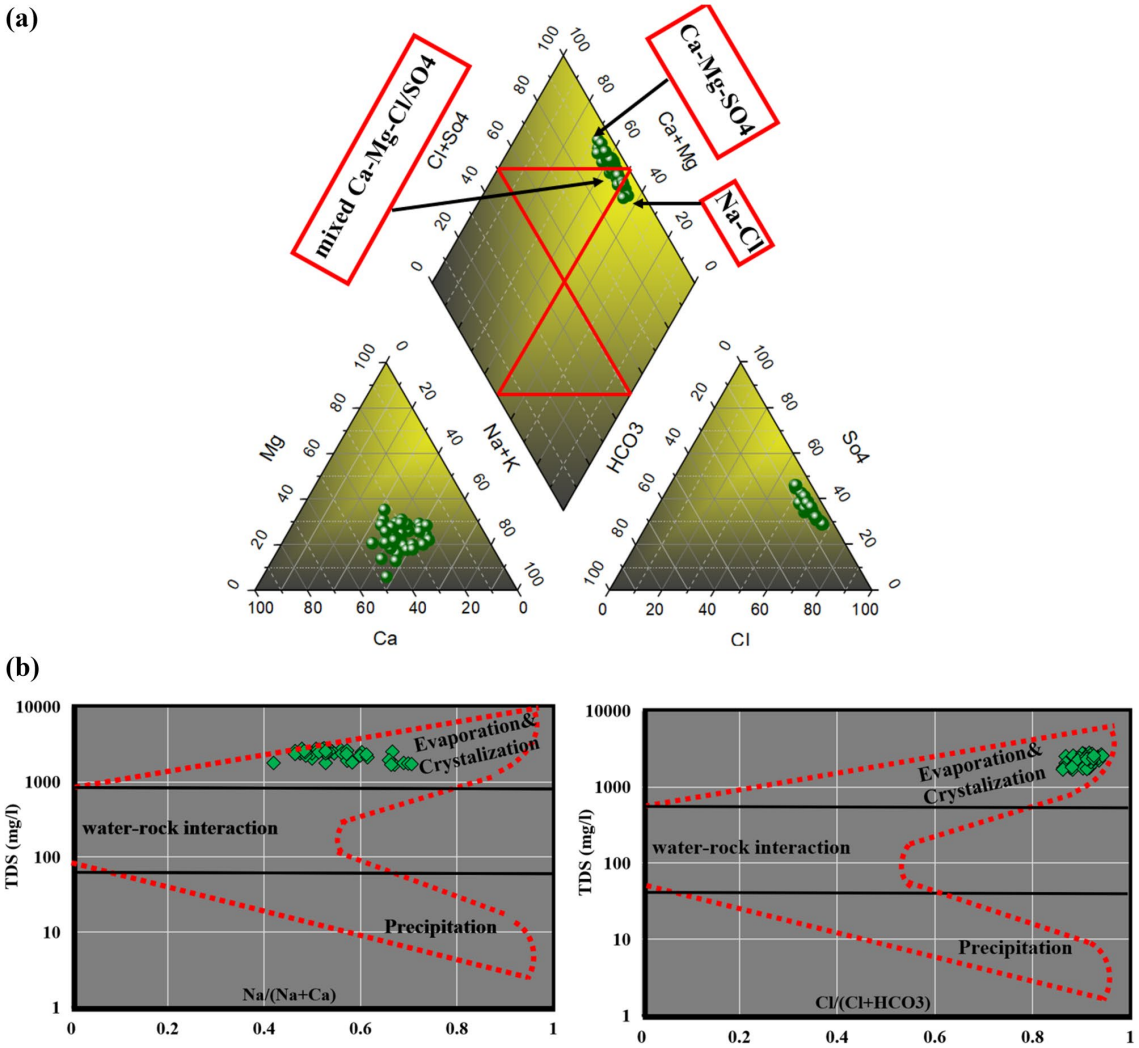
Piper plot (**a**) illustrates the water type/facies of all samples from different locations in Oued-Souf, and Gibbs Plot (**b**).

A Piper plot, originally proposed by Piper^81^, was utilized to categorize the water type/facies observed in water samples (Fig. 3a). The collected samples were categorized into three distinct facies on the Piper scatter plot (Fig. 3a). Thirty-one samples were found to fall within Ca – Mg – SO_4_ facies zone, characterized by permanent hardness potentially attributed to ion exchange (reverse type) processes. The salinity of such water was notably high, primarily due to elevated concentrations of calcium and chloride, particularly evident in the Debila region. Few samples (Six samples) mainly from El-Oued and Debila were classified under the Na–Cl facies, indicative of the evaporation process serving as the primary factor govern water evolution in the investigated area. The remaining samples fell into the mixed Ca–Mg–Cl/SO_4_ class zone, dispersed across three parts of investigated region (Debila regions, Hassi Khalifa, and El-Oued).

**Figure 4:**
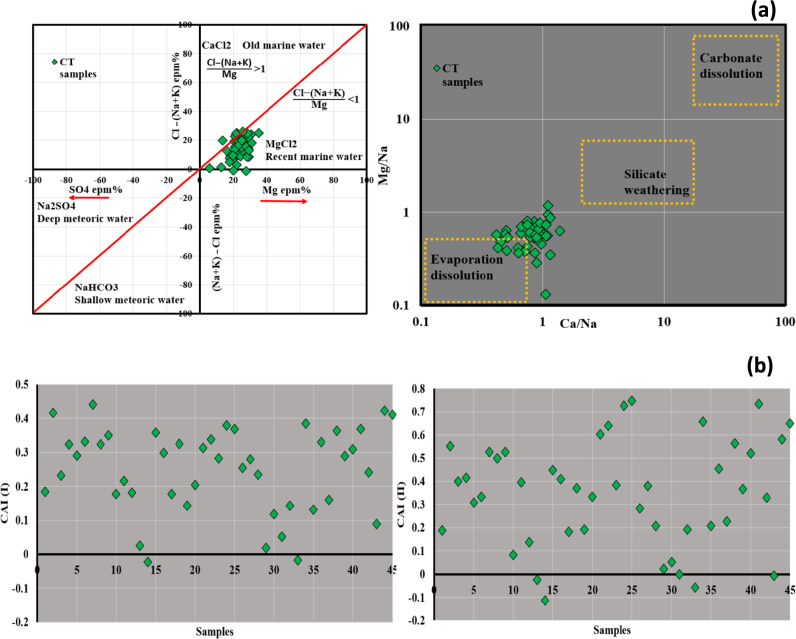
Sulin scatter plot and ionic ratios Mg^2+^/Na^+^ vs Ca^2+^/Na^+^ showing water origin mechanism control water chemistry in CT aquifer (**a**), type of ion exchange using CAI (**b**).

The Gibbs diagram's scatter plot^82^ is a widely used tool for elucidating the influence of various mechanisms on water chemistry and delineates the diagram into three primary zones^1,83^ (Fig. 3b). Examination of the graphical plot indicates that all samples are situated inside the domain of evaporation/crystallization. To further corroborate that the prevailing mechanism shaping the chemistry of water within the CT aquifer is evaporation crystallization/dissolution, the log ionic ratio of Mg/Na versus Ca/Na **(**Fig. 4a**)** was employed.

The ionic ratio showed that the majority of samples fell between evaporation dissolution and silicate weathering zones referring to the two main mechanisms controlling the GW chemistry in Oued-Souf area. The Sulin graph^84^ (Fig. 4a) is an effective tool to detect the groundwater origin and differentiate between deep meteoric, shallow meteoric, old marine, and recent marine water based on the percentage of (Cl-(Na + K))/Mg and Cl-(Na + K) values in epm%. In the current research, the water samples fell in the marine origin, specifically in recent marine origin with MgCl_2_ in composition. Sulin diagram has some limitation in case of evaporation dissolution/ crystallization is the primary process governing the water chemistry which shift the plots to marine origin instead of meteoric origin specially when there are marine sediments such as dolomite and calcite which is the case in Oued-Souf area. The isotopic tracers could confirm the paleo meteoric origin of the CT from previous study^85^. These results showed that Sulin diagram alone is not suitable to detect the origin of the groundwater specially in the presence of marine sediments and the controlling mechanism is evaporation dissolution. There was also similar case study in the Siwa Oasis, Egypt showed the same limitation of Sulin diagram^7^.

The ion exchange is not excluded in the study area where the aquifer composition contains silicate minerals and carbonate minerals. There are two types of reactions decide if the water is influenced by direct ion exchange or reverse ion exchange based on the chloro-alkaline indicators (CAI-I and CAI-II) by implementing the concentration of Cl^−^, Na^+^, CO_3_^2−^, HCO_3_^−^, SO_4_^2−^, and NO_3_^-^. These indices are very effective tool demonstrate the water rock interaction through replacement of ions such as Ca^2+^, and Mg^2+^ with Na^+^ and K^+^. In the current study, the CAI-I and CAI-II results (Fig. 4b) exhibited that most samples represented by positive value greater than zero which reveal that the reverse ion exchange is a significant mechanism controlling the water chemistry of the CT aquifer. The reverse ion exchanges replace the calcium and magnesium in rocks with sodium and potassium in water.

$$\begin{aligned} & 1/2{\text{Ca}}^{2 + } - {\text{X}} + {\text{Na}}^{ + } \to 1/2{\text{Ca}}^{2 + } + {\text{Na}}^{ + } - {\text{X}}\quad ({\text{Reverse}}\,{\text{ion}}\,{\text{exchange}}) \\ & {\text{Na}}^{ + } - {\text{X}} + 1/2{\text{Ca}}^{2 + } \to {\text{Na}}^{ + } \, + \,1/2{\text{Ca}}^{2 + } - X\quad ({\text{Direct}}\,{\text{Ion}}\,{\text{exchange}}) \\ \end{aligned}$$

Piper plot was performed using Diagrammes software, while Gibbs and ion ratio plots were created using Excel file.

### Geochemical modeling and ion source detection

**Figure 5:**
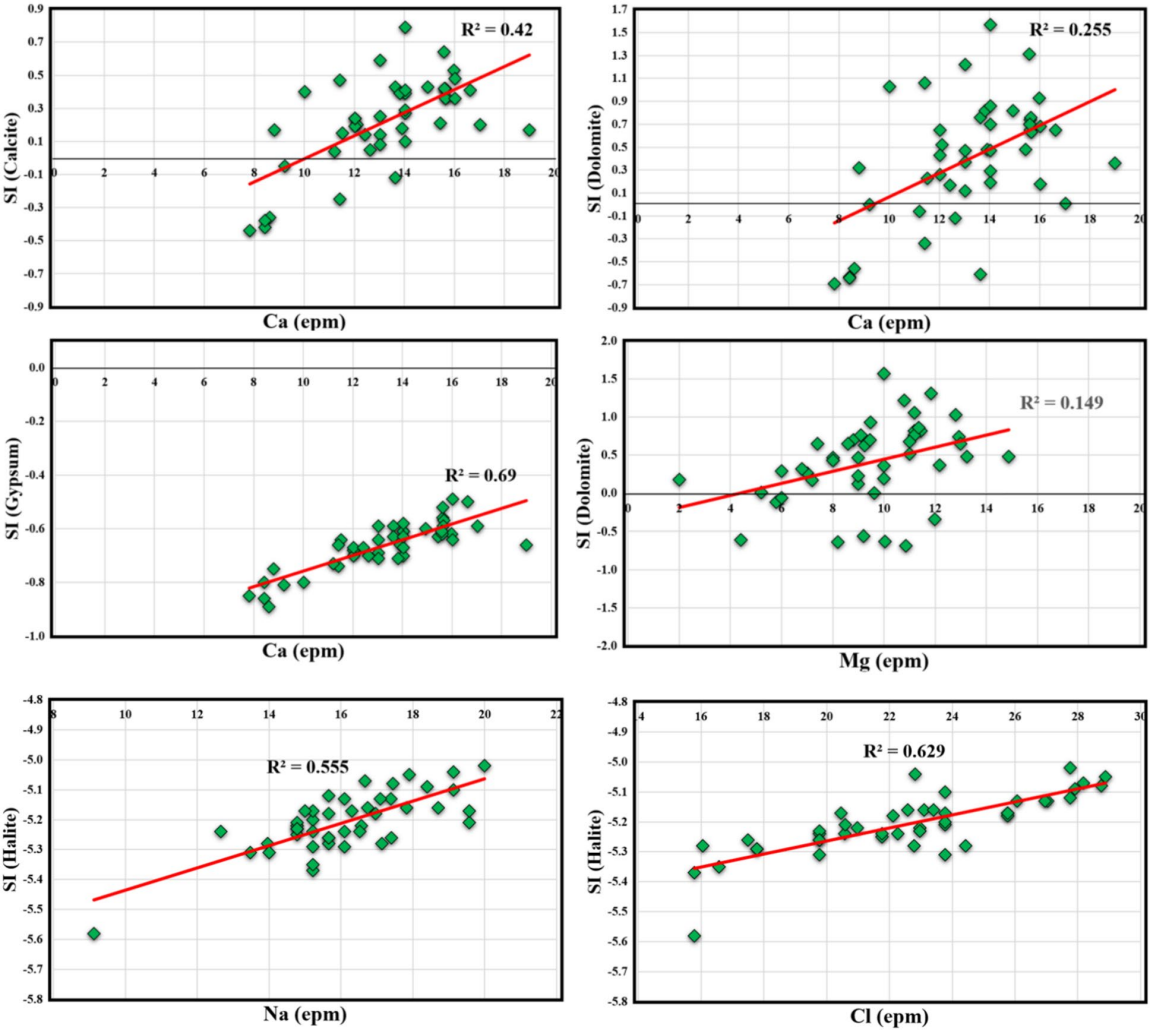
Geochemical modeling using PHREEQC to detect the minerals saturation state and the relationship between the ions and the saturation index (SI) of different minerals.

The geochemical modeling was performed using PHREEQC to detect the minerals saturation state and the relationship between the ions and the saturation index (SI) can determine the main contribution minerals in the aquifer system that could increase the concentration of the Calcium, magnesium, Chloride, and sodium (Fig. 5). The hypnoses of the model depend on input physical, and chemical attributes or parameters and the output is the saturation index of calcite, gypsum, dolomite, and halite. To make sure that the simulated minerals is accurate, the temperature and pH values measured in the field were used to represent the true state of the aquifer condition where the mineral saturation is sensitive to the physical parameters. Based on the current results and the ionic ratio with between the input and output parameters, The groundwater collected from CT were super saturated with dolomite and calcite which indicates the possibility of water to precipitate these minerals during the irrigation process. The precipitation of these minerals in the soil can decrease the infiltration rate and create water logging problem and decline the plant production. It is not recommended to use calcium fertilizers in the study area to do not deteriorate the physical and chemical structure. On the other hand, gypsum and halite minerals showed very low value of SI (negative value) which indicates the capacity of water to dissolve more from these minerals and the aquifer system does not contain high quantity of these minerals which can be confirmed from the relationship between ions and SI. The relationship of Ca vs calcite, gypsum, and dolomite showed week correlation with dolomite (R^2^ = 0.25) and calcite (R^2^ = 0.42) and high correlation with gypsum (R^2^ = 0.69) which indicate that the main contributors for Ca^2+^ in the CT could be dissolution of gypsum and silicate minerals weathering or reverse ion exchange. The high concentration of SO_4_^2−^ confirm the significant contribution of gypsum dissolution. The week correlation between Mg^2+^ and dolomite reveal that the presence of magnesium ions in the CT aquifer mainly come from silicate weathering or reverse ion exchange. There is moderate to high correlation of Na^+^ (R^2^ = 0.55) and Cl^−^ (R^2^ = 0.63) with halite minerals which indicate the geogenic and anthropogenic sources could have strong effect to increase the concentration of Na^+^ and Cl^-^. The current research findings suggest treatment of water is required before irrigation process to avoid soil salinization and plant reduction.

Previous global research studies used geochemical modeling, ionic ratios, and statistical analysis to detect the source of ions and minerals in different water resources in arid, wet, and semi-arid countries due to the water rock interaction and anthropogenic sources^1,6,7,14,15,31,86^. Several researchers reported that the PHREEQC model and saturation indices were effective tools to detect the minerals saturation in water reflecting the geological composition without the need for solid samples^1^ and the risk of supersaturation of some minerals on the soil permeability and increasing water logging problems^86^. Based on groundwater samples collected from the Al-Jawf basin in Yemen, the Debrecen aquifer in Hungary, and the Nubian aquifer of Siwa Oasis in Egypt, it was noted that the source of Ca, Mg, Na, could originate from silicate weathering and form calcite and dolomite minerals with supersaturation state, where the aquifer system mainly composed of silicate minerals with some carbonate (calcite and dolomite) and evaporites minerals (gypsum and Halite) increase the concentration of Na^+^, Cl^-^, SO_4_^2−^, Ca^2+^, and Mg^2+^ through mineral or salt dissolution^1,6,15^ Which could be confirmed by ionic ratios and correlation matrix in central Odisha of India^31^.

### Principal component and Cluster analysis

**Figure 6:**
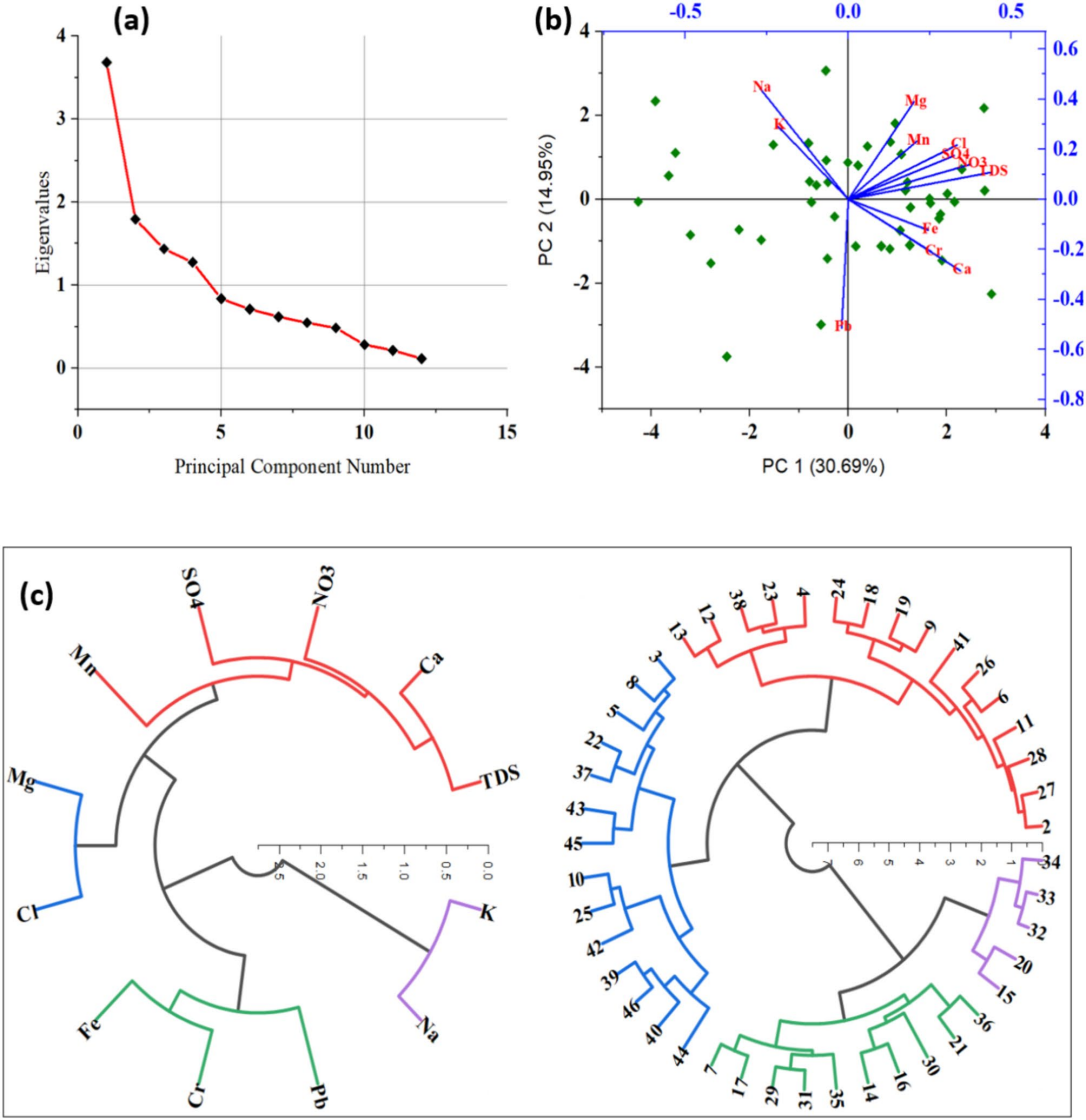
The extracted components from PCA based on scree plot (**a**) and PCs in 2D plot (**b**), and cluster analysis using Dendrogram circle showing the correlation between ions (**c**).

The Kaiser–Meyer–Olkin analysis (≥ 68) and Bartlett's Sphericity test (P < 0.05) validate the suitability of the water quality dataset for PCA, indicating adequate inter-variable relationships. The eigenvalues were higher than 1 (Table 3) which proof the optimum number of components extracted is acceptable for interpretation of the datasets (Fig. 6a).

**Table 3:** The three extracted components (PC1, PC2, and PC3) from PCA using physico-chmical and PTEs parameters.

Parameters	PC1	PC2	PC3	PC4
TDS	0.44	0.11	− 0.15	0.11
Ca^2+^	0.34	− 0.29	− 0.41	0.13
Mg^2+^	0.20	0.39	0.48	− 0.25
Na^+^	− 0.27	0.44	0.13	0.42
K^+^	− 0.22	0.29	− 0.24	0.57
Cl^-^	0.33	0.22	0.20	0.10
SO_4_^2−^	0.32	0.17	0.01	− 0.10
NO_3_^−^	0.37	0.14	0.08	0.00
Fe	0.25	− 0.12	0.36	0.41
Mn	0.21	0.23	− 0.47	− 0.07
Cr	0.26	− 0.21	0.05	0.44
Pb	− 0.02	− 0.51	0.33	0.15
Eigenvalue	3.68	1.79	1.44	1.28
Percentage of Variance	30.69%	14.95%	11.97%	10.63%
Cumulative	30.69%	45.64%	57.62%	68.24%

PC1, accounting for 30.69% of the variance, shows a correlation between TDS, Ca^2+^, Cl^−^, SO_4_^2−^, and NO_3_^−^. This suggests that these variables may share a common source or underlying relationship in the context of water quality. PC1 can represent the water rock interaction and dissolution process of gypsum and halite minerals are the main reason for increasing the salinity of water in the aquifer system. PC2, explaining 14.95% of the variance, demonstrates a correlation between Na^+^ with Mn, implying that these elements may originate from a similar source or geological process. PC3, contributing 11.97% of the variance, shows Mg^2+^ standing alone with Pb, indicating that it might have a distinct influence or source compared to the other variables in the dataset. PC2 represent the anthropogenic and geogenic source which confirm the previous geological modeling that there is another source of Na and Mn ions rather than halite and dolomite dissolution. Lastly, PC4, explaining 10.63% of the variance, reveals a correlation between Fe, Cr, and Pb. This association suggests that these variables might be influenced by similar environmental factors or contamination sources which is mainly from anthropogenic activities (Fig. 6b).

The Ward's method and dendrogram analysis revealed the formation of four distinct clusters based on the similarities in the water quality variables. The observed correlations among total dissolved solids (TDS), calcium (Ca^2+^), nitrate (NO_3_^-^), sulfate (SO_4_^2−^), and manganese (Mn) propose a shared source or common processes influencing their presence in the water samples. One plausible explanation for this correlation could be geological factors, wherein the composition of the underlying bedrock or soil influences the leaching of minerals into the water. For instance, high levels of calcium and sulfate are commonly associated with carbonate-rich geological formations, while manganese may originate from weathering of manganese-bearing minerals present in the surrounding environment. The correlation with nitrate could be indicative of agricultural runoff or anthropogenic inputs such as fertilizers, which contribute to elevated nitrate levels in water bodies. Similarly, the correlation between magnesium (Mg^2+^) and chloride (Cl^-^) underscores potential shared sources or environmental processes governing their concentrations. Chloride and magnesium ions are often associated with mineral dissolution or anthropogenic inputs. The association between potassium (K^+^) and sodium (Na^+^) suggests common sources (silicate weathering or anthropogenic activities) or transport mechanisms affecting their presence in the water samples. Both K^+^ and Na^+^are common constituents of agricultural fertilizers, and their correlation could reflect agricultural runoff or irrigation practices in the watershed. Furthermore, the correlation observed among iron (Fe), chromium (Cr), and lead (Pb) warrants attention due to their potential environmental and health implications. These metals are often associated with industrial activities, mining operations, or urban runoff, where anthropogenic sources play a significant role in their introduction into water bodies in the investigated area. The clustering of these metals underscores the importance of monitoring and mitigating sources of pollution to prevent adverse impacts on environment and human health (Fig. 6c).

### Groundwater suitability for drinking using IWQI

**Figure 7:**
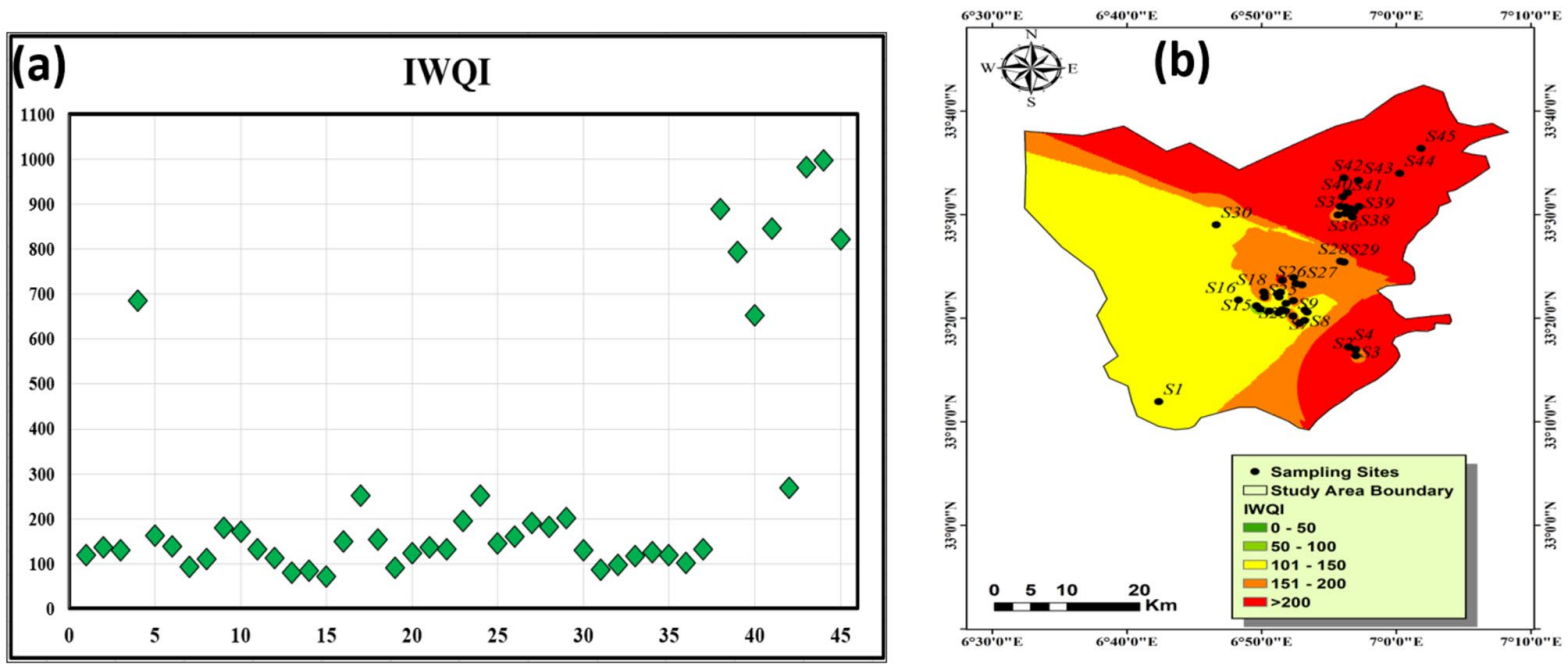
The calculated IWQI and plotting its values for all samples (**a**) and IWQI distribution map in Oued-Souf area (**b**).

The water quality index for consumption (drinking) purposes based on IWQI could be classifies into four main quality type (extremely poor, poor, medium or intermediate good, and excellent quality) according to the calculated values. The range of this classification is > 200, 150–200, 100–150, 50–100, and 0–50 for extremely poor, poor, medium or intermediate good, and excellent quality respectively. In the current study, the IWQI ranges from 71.4 to 998.9 with an average value 277. Figure 7a classifies water based on IWQI values, indicating that 26.6% of water samples fell into the extremely poor category (IWQI > 200) including all water samples located in Debila, Hassi Khalifa, Baydah, and Robbah area (Fig. 7b). The results showed that 17.7% of samples had IWQI values between 150 to 200 which fell within poor quality range represented by 8 samples collected from northern part of El-Oued. The IWQI value in 40% of samples raged from 100 to 150 including 18 samples from Gummer, and southern part of El-Oued which indicates medium quality category. The rest of samples (15.5% of samples) fell within good to excellent quality represented mainly by El-Oued samples (S7, S13, S14, S15, S19, S31, and S32). The current findings demonstrated that the groundwater (GW) of the CT aquifer in the central and southern El-Oued region is the most appropriate for drinking based IWQI, while the other parts of the investigated area need further treatment and suitable management to prevent any health risks regarding the water quality. The drinking water quality of the CT deteriorate from west to east direction based on the interpolation of the IWQI values in the study area. The study area, primarily agricultural, involves extensive crop cultivation with fertilizer application and significant animal husbandry activities. The main reason for the quality deterioration of the CT is increasing the salinity due to water rock interaction and over extraction of water for irrigation purposes as well as return back of agricultural drainage through the leakage downward.

A variety of strategies have been used to assess groundwater quality, including the fuzzy comprehensive evaluation method^60,87^ and the matter-element extension method^88^. The entropy-based weighted technique is used to calculate the weights of hydrochemical parameters in groundwater and assess their quality. This method aids in minimizing the examination of excessive components, precisely defining water quality categories, and establishing if the assessed variables are consistent with decision-making criteria for some functional regions^89,90^. The entropy-weighted water quality index (EWQI) calculates groundwater quality precisely, allowing it to be ranked according to groundwater quality criteria^31,89,91^.

The current results demonstrated that the parameter's contribution to the drinking groundwater quality and increasing IWQI values are arranged in descending order based on the calculated integrated weight as follows Mn > NO_3_ > Fe > K > HCO_3_ > Cl > Na > SO_4_ > Ca > Mg > pH > TDS > EC > TH. These findings recommend further treatment of the groundwater before consumption, considering the calculated weight of the current research for sustainable water management.

### Heavy metal pollution index (HPI) and Heavy Metal Index (HMI)

**Figure 8:**
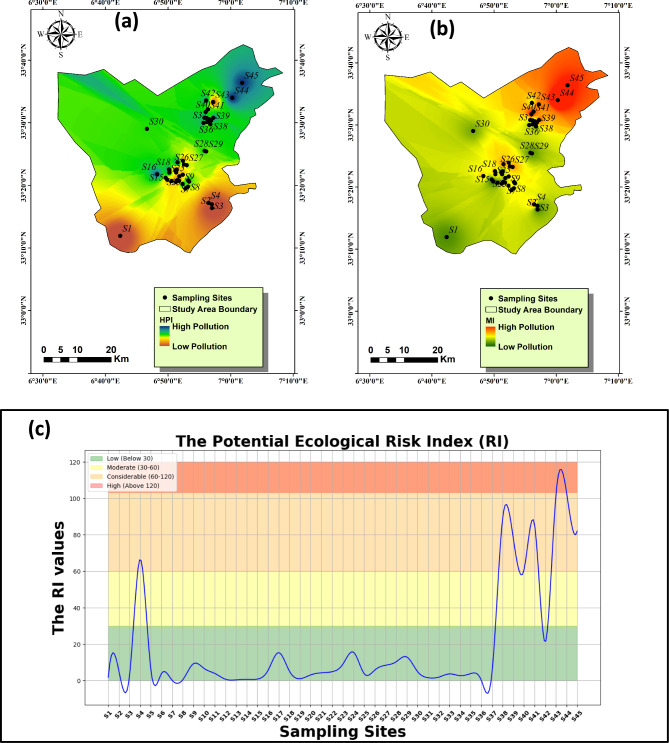
Distribution map of the PTEs indices including HPI (**a**) and MI (**b**) and ecological risk values calculated from the PTEs with each sample (**c**) in Oued-Souf.

The utilization of the heavy metal pollution model (HPI) constitutes a fundamental approach for comprehensively calculating the pollution levels within surface and groundwater environments. The utilization of this model facilitates the evaluation of PTEs impacts on freshwater quality, thereby enabling effective monitoring and management strategies to mitigate potential risk linked to with PTEs exposure^7^. Notably, the mean HPI value during the period of observation recorded an average of 240, demonstrating a range spanning from 46.09 to 410.28. These results underscore the substantial presence of heavy metal contaminants in the groundwater samples collected within the study period. According to Edet and Offiong^66^ classification, nearly 16% of the samples fell within the low pollution category (HPI < 100), indicating relatively lower levels of heavy metal contamination. However, the majority, constituting 84% of the samples, exhibited high pollution levels (Fig. 8a), reflecting a concerning prevalence of heavy metal pollutants in the groundwater. Therefore, there are several important considerations regarding the underlying factors driving heavy metal pollution in the study area. Environmental factors such as industrial activities, urbanization, and agricultural practices may contribute to the influx of heavy metals into groundwater bodies, exacerbating pollution levels. Additionally, natural processes such as weathering and erosion can also influence the mobilization and transport of heavy metal contaminants, further exacerbating the issue.

To deepen our understanding of heavy metal impact on water quality, we employed the Metal Index (MI) methodology across multiple sampling sites. This method measures the extent of heavy metal presence in water, utilizing threshold values outlined in the WHO guidelines as benchmarks for acceptability. Based on the data of heavy metal indix, several sampling sites stand out for their concerning levels of pollution. Specifically, sites S3, S14, S19, S21, S31, and S36 exhibit considerable pollution (Fig. 8b), indicating elevated concentrations of heavy metals. These findings suggest a potential environmental risk in these areas, necessitating further investigation and remedial actions to mitigate the pollution and protect environmental and human health. Additionally, sites S1, S2, S11, S13, S15, S22, S28, and S37 are identified as heavily polluted, indicating a significant presence of heavy metals above acceptable thresholds (Fig. 8b). Urgent attention is required to address the pollution in these locations and prevent further degradation of the environment. Meanwhile, the remaining sampling sites are classified as severely polluted, highlighting the widespread nature of heavy metal contamination across the sampled area. Comprehensive measures are essential to address these pollution levels effectively and protect the environment for current and future generations.

## Ecological risk index (RI)

The Possible Ecological Risks Indicator, devised by Hakanson^69^, stands as a commonly acknowledged approach utilized to measure the degree of PTEs pollution and its plausible ramifications on both sedimentary and aquatic environments. This indicator considers various parameters, encompassing heavy metal concentrations, their toxicological and ecological effects, as well as greater environmental effects. This work focused on the ecological risk indicator (RI) of PTEs (Fig. 8c) in groundwater of Oued-Souf aquifer. The computed average RI value for the investigated samples stood at 18.99, ranging from 0.03 to 103.21. These observations indicate that a substantial portion, constituting 82% of the samples, pose low RI value (RI < 30); however, the remaining 18% signify a noteworthy environmental pollution risk. Figure 8c delineates the ecological risk indicator concerning the sampling locations. This visual representation elucidates the nexus between pollution levels and the ensuing ecological risk. The investigated area is characterized by considerable agricultural and residential sectors proximate to the basin and its tributaries. These factors potentially contribute to the accrual of heightened metals amounts in sedimentary deposits, subsequently infiltrating into the study area. Hence, the evaluation of prospective ecological hazards linked with heavy metal contamination in aquatic ecosystems holds paramount significance.

### Human health risk assessment

The evaluation of non-carcinogenic and carcinogenic risk hazard indices involved the computation of hazard quotients (HQ) for ingestion and dermal absorption pathways. These results unveil the collective health risks posed to both adults and children due to exposure to various heavy metals.

### Non-carcinogenic health risk

**Figure 9:**
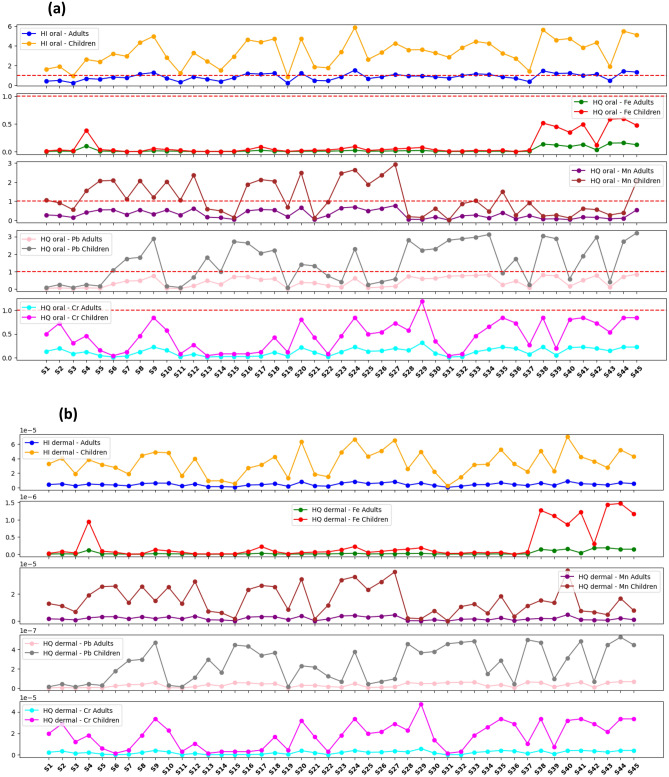
The estimated risk indices in each metal (HQ) and combined metals (HI) through different route of exposure in adult and child.

The hazard quotient (HQ) values for oral exposure to heavy metals, including Mn, Fe, Cr and Pb were assessed for both adults and children. For adults, the HQ values ranged from 4.31E−05 to 1.56E−01 for Fe, from 3.77E−03 to 7.92E−01 for Mn, from 1.00E−02 to 3.11E−01 for Cr, and from 2.15E−02 to 8.40E−01 for Pb. Meanwhile, children exhibited higher HQ values across all metals, with ranges from 1.64E−04 to 5.94E−01 for Fe, from 1.44E−02 to 3.03E+00 for Mn, from 3.84E−02 to 1.19E+00 for Cr, and from 8.22E−02 to 3.21E+00 for Pb. In comparison, for hazard index (HI) through oral/ingestion, adults exhibited HI values ranging from 2.31E−01 to 1.54E+00, while children showed higher values, ranging from 8.84E−01 to 5.88 E+00 (Fig. 9a). These results underscore varying degrees of health risks connected with heavy metal exposure, with children consistently showing higher HQ values across all metals, highlighting the increased vulnerability of children to heavy metal toxicity. Such findings emphasize the critical need for targeted interventions to reduce exposure and protect public health, particularly among children.

On the other hand, the hazard quotient (HQ) values for dermal exposure to heavy metals Fe, Mn, Cr, Pb were assessed for two age groups (adults and child). In adults, HQ values for Fe ranged from 5.1E−11 to 1.8E−07, while for children, they varied from 4.1E−10 to 1.5E−06. For Mn, adults exhibited HQ values ranging from 2.2E−08 to 4.6E−06, whereas children displayed values between 1.7E−07 to 3.7E−05. Chromium showed HQ values ranging from 1.8E−07 to 5.85494E−06 in adults and from 1.5E−06 to 4.7E−05 in children. Lead exhibited HQ values ranging from 1.7E−09 to 6.5E−08 in adults and from 1.4E−08 to 5.3E−07 in children. Additionally, HI for dermal exposure showed values in adults ranging from 2.7E−07 to 8.7E−06 and in children from 2.2E−06 to 7.1E−05. These findings underscore the possible risks associated with dermal exposure to PTEs (Fig. 9b).

### Monte Carlo simulation approach

Monte Carlo simulation was used to estimate the hazard quotient (HQ) values for oral and dermal exposure to of Fe, Mn, Cr, and Pb, as well as the cancer risk (CR) from oral and dermal exposure to Cr and Pb, for adult and child.

### Non-carcinogenic risk

**Figure 10:**
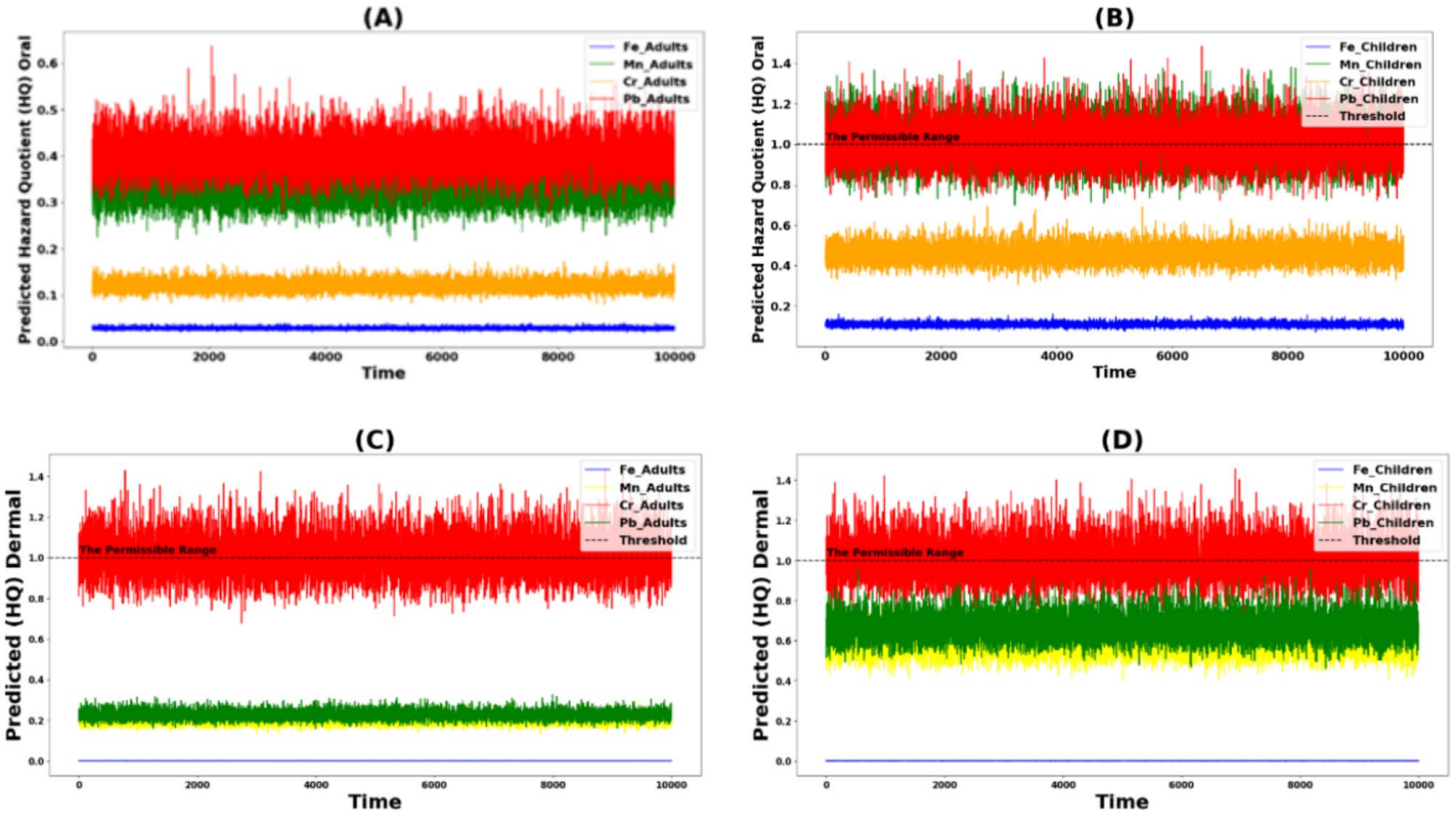
The Hazard quotient simulated from Monte Carlo in all groups with two different ways of exposure.

The Monte Carlo simulation results indicated that the predicted oral hazard quotient (HQ) values for adults for all assessed heavy metals (Fe, Mn, Cr, and Pb) remained below the standard safety threshold (HQ < 1) (Fig. 10a), suggesting a manageable risk level for the adult population. However, for children, the oral HQ values for manganese (Mn) and lead (Pb) surpassed the threshold (HQ > 1) (Fig. 10b), highlighting a significant health risk and the need for urgent attention to reduce exposure levels in this vulnerable group. Additionally, the dermal HQ values for chromium (Cr) in both adults and children exceeded the standard limits (HQ > 1) (Fig. 10c, d), indicating a critical risk associated with dermal exposure to chromium. However, it's important to recognize that risk assessments often rely on conservative assumptions and uncertainties in existing data. Therefore, continuously monitoring exposure levels and updating risk assessments with new information as it becomes available is crucial^20^.

### Comparison with previous studies

Some researchers used a similar approach to evaluate the health risk regarding several parameters in water with different aquifer and geological conditions and proved the efficiency of using Monte-Carlo simulation to predict the reliable risk with low uncertainty^40^. Gao^40^ Studied the risk of F^-^, NO_3_^-^, Cr (VI), and As in 321 drinking groundwater samples collected from Guanzhong Basin (GB) in China and referred to the urgent need for this investigation where the children were more vulnerable to these elements than adults through oral/ingestion exposure route. Gao confirmed that there is a possibility, around 92.25%, that children may encounter health risks that are not related to cancer. An analysis of sensitivity revealed that the primary health hazards for inhabitants stem from concentrations of F^−^ and Cr^6+^ in the groundwater^40^. Studies on hydrogeochemistry have suggested that the substandard quality of groundwater in GB is mainly due to the region's characteristics and extensive evaporation caused by prolonged irrigation methods. In the current study area (Oued-Souf), the non-carcinogenic risk regarding oral exposure originated mainly from the Fe and Mn. Future work could include the health risks regarding Fluoride, arsenic, and nitrates.

### Carcinogenic health risk

In our research, we employed the Monte Carlo technique to model carcinogenic risk evaluations derived from the deterministic approach, considering dermal and oral exposure routes in both children and adults’ communities. The 95th and 5th percentile risk exposures were indicative of the most favorable and most adverse scenarios, respectively.

#### Lead

**Figure 11:**
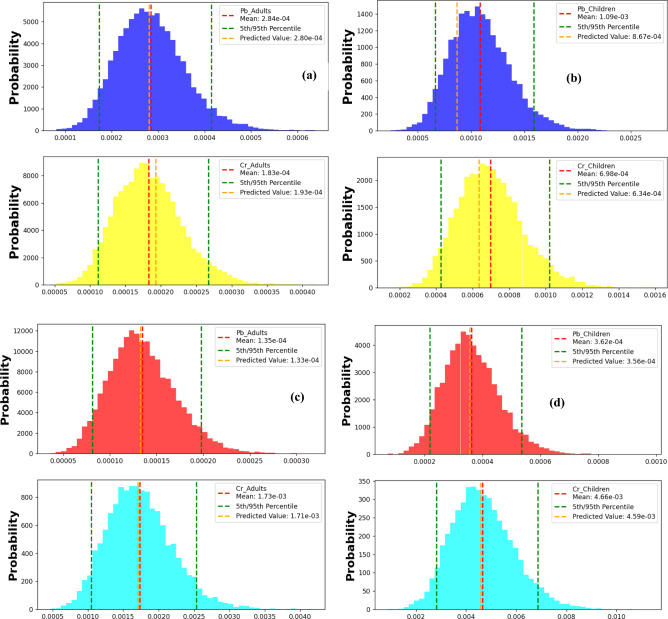
The CR index simulated for Cr and Pb from Monte Carlo in all groups with two different ways of exposure.

Histograms represented in Figs. 11 a-d were generated employing 10,000 epochs to simulate the carcinogenic risk associated with lead exposure via ingestion and dermal routes in both adult and children’s populations. The data analysis indicates a heightened risk of cancer development in children compared to adults, with their ingested CR levels surpassing the threshold value of 1.0E−04 (oral CR 95% = 1.7E−03), whereas for adults, it slightly exceeded the threshold at 4E−04. Conversely, for dermal exposure, the mean CR values were recorded as 1.35E−04 for adults and 3.62E−04 for children, respectively. Notably, at the 95th percentile, the dermal CR levels for adults (2.1E−04) and children (5.3E−04) both exceeded the threshold, indicating susceptibility to cancer via dermal exposure in both age groups. Hence, the reduction of lead amounts in the environment emerges as imperative in mitigating the correlated health risks, particularly concerning children^20^.

#### Chromium

Figures 11 a-d illustrate histograms portraying the CR resulting from chromium exposure via oral and dermal routes in both adult and children’s populations. The mean CR values for oral exposure suggest a significantly heightened vulnerability among children compared to adults, with children exhibiting a six-fold increase in cancer susceptibility. This is evidenced by the oral CR score/level surpassing the threshold value of 1.0E−04, where the 95th percentile oral CR for children reached 1.0E−03, while for adults it was slightly lower at 2.5E−04. Conversely, the CR results for dermal exposure at the 95th percentile indicate susceptibility to cancer in both children and adults. Specifically, children displayed a CR of 6.8E−03, while adults exhibited a CR of 2.5E−03. Consequently, mitigating environmental chromium levels holds promise for reducing the associated health risks linked to exposure^20,92^.

## Conclusion

This investigation evaluated the ecological and health issues associated with potential toxic elements (PTEs) in the complex terminal (CT) aquifer of the Algerian desert. The assessment utilized principal component analysis (PCA) and cluster analysis (dendrogram) to identify pollution sources and quality-controlling factors. Various indices (HPI, MI, HQ, HI, and CR) were used to evaluate environmental and human health risks. Additionally, the Monte Carlo method was applied for probabilistic carcinogenic and non-carcinogenic risk assessment through oral and dermal exposure routes in adults and children. Approximately 16% of the samples fell within low pollution class (HPI < 100), indicating relatively lower heavy metal contamination levels. However, 84% of the samples showed high pollution levels, reflecting significant heavy metal contamination in the northeastern part of the study area. The average RI values for the samples were 18.99, ranging from 0.03 to 103.21. This indicates that 82% of the samples pose low ecological risk (RI < 30), whereas 18% present a notable environmental pollution risk. The hazard index (HI) for oral ingestion in adults ranged from 2.31E−01 to 1.54, and in children, it ranged from 8.84E−01 to 5.9 (Fig. 5a). For dermal exposure, HI values in adults ranged from 2.71E−07 to 8.74E−06, and in children, from 2.18E−06 to 7.03E−05. These findings highlight the potential non-carcinogenic risks associated with oral exposure to PTEs and the increased vulnerability of children to Fe, Mn, Pb, and Cr. Most samples had CR range exceeding 1 × 10^–4^ for the two metals (Cr and Pb), indicating a carcinogenic risk for both age groups. Monte Carlo simulations confirmed these findings, showing a significant carcinogenic impact on both children and adults. The simulation results specified that the predicted oral hazard quotient level (adults) for all assessed metals (Fe, Mn, Cr, and Pb) remained below the standard safety threshold (HQ < 1), suggesting a manageable risk level for adult group. However, for children group, the oral HQ values for manganese (Mn) and lead (Pb) exceeded the threshold (HQ > 1), highlighting a significant health risk and the urgent need to reduce exposure levels in this vulnerable group. Additionally, the dermal HQ values for chromium (Cr) in both adults and children exceeded standard limits (HQ > 1), indicating a critical risk from dermal exposure to chromium. These findings emphasize the urgent need for targeted interventions to reduce exposure and protect public health, especially among children.

### Supplementary Information

Supplementary Information.

## Data Availability

The datasets utilized and/or analyzed during the current study are available upon request from the corresponding author.
