# Supplementary material for: Advanced approach combines integrated weight water quality index and potential toxic elements for environmental and health risk assessment supported by simulation technique in Oued Souf, Algeria
Source: Sci Rep. 2024 Aug 1;14:17805. doi: 10.1038/s41598-024-68854-1 (PMC11294618; doi:10.1038/s41598-024-68854-1)
Supplement: Supplementary file 1 — Supplementary Information. [file 41598_2024_68854_MOESM1_ESM.docx]

**Advanced approach combines integrated weight water quality index and potential toxic elements for environmental and health risk assessment supported by simulation technique in Oued Souf, Algeria**

| 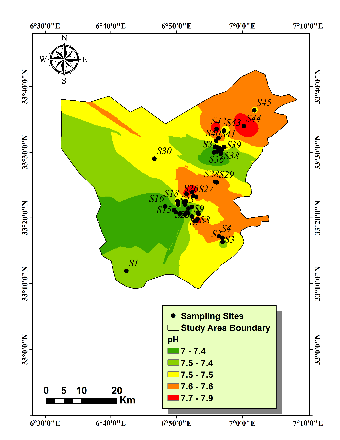  a | 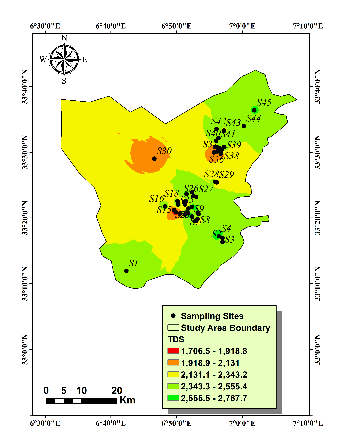  b | 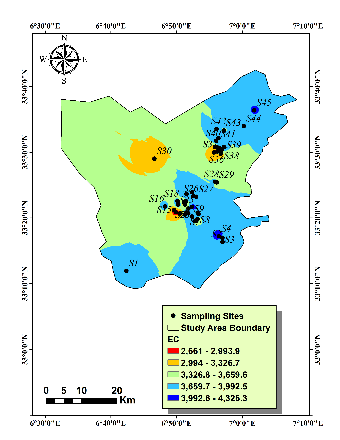  c | 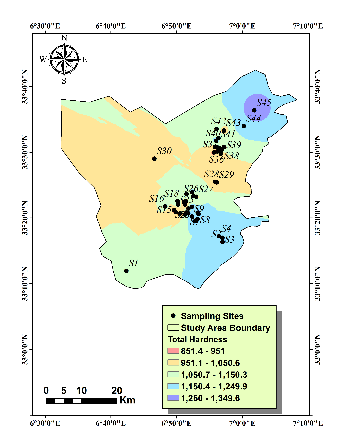  d |
| --- | --- | --- | --- |
| 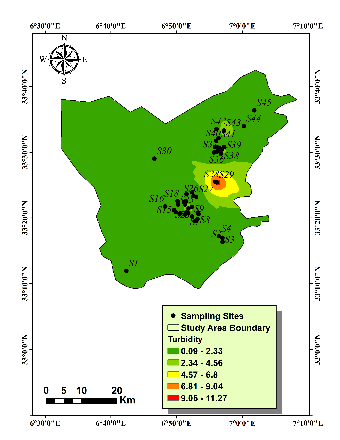  e | 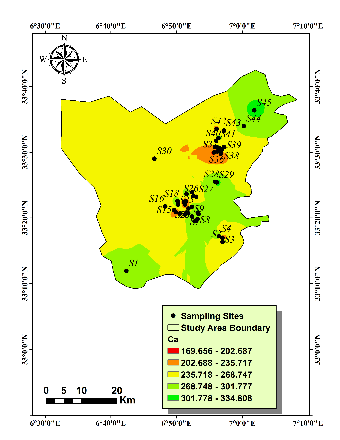  f | 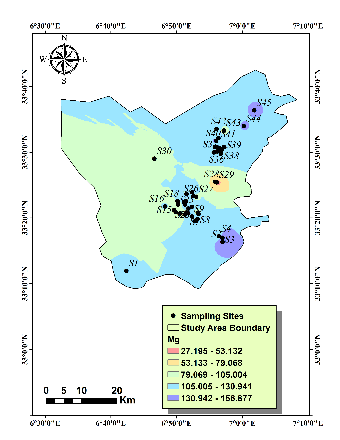  g | 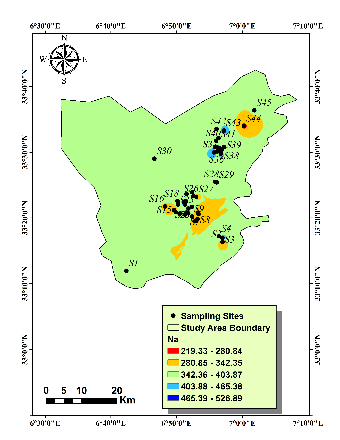  h |
| 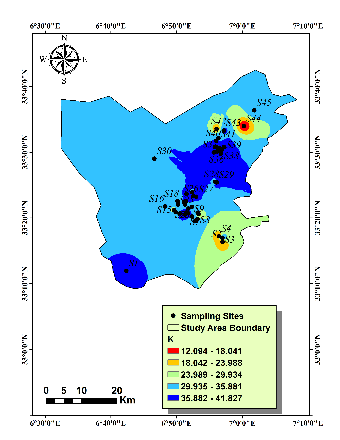  i | 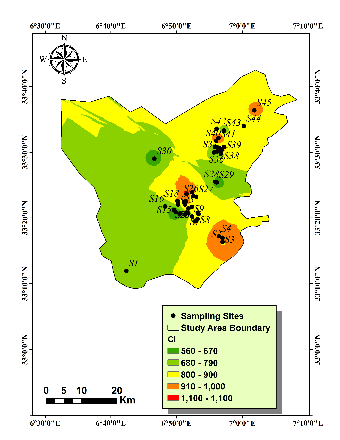  j | 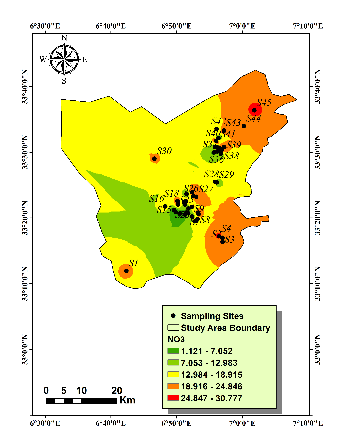  k | 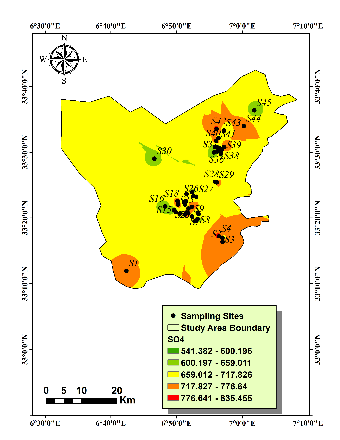  l |
| 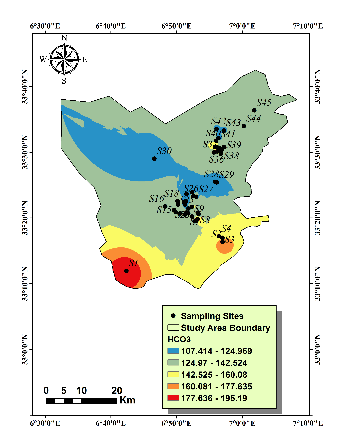  m | 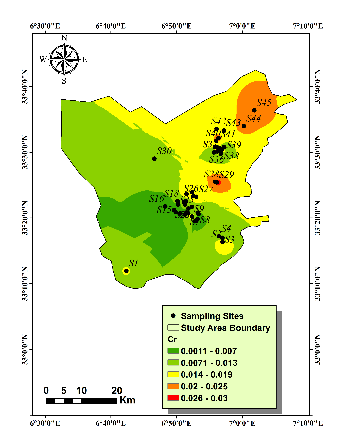  n | 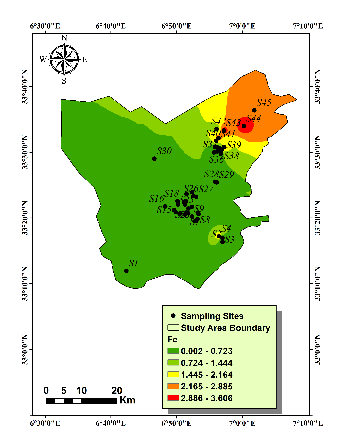  o | 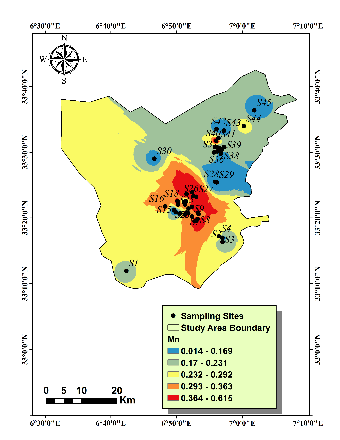  p |
| 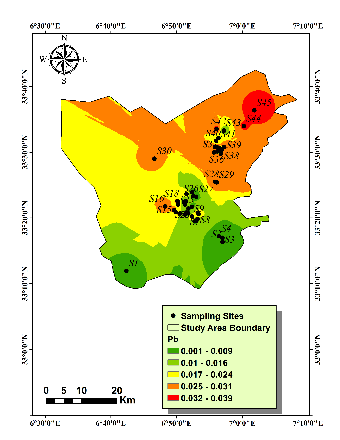  q |  |  |  |

**Figure S1.** Distribution maps of all measured parameters to detect the most location could be affected by water quality deterioration.

Table S1. The parameters used to calculate HQ, HI, and CR

| HM | RfD Oral(mg/kg/day) | ABS | Rfd Dermal(mg/kg/day) | CSFing mg/kg/day | CSFder | Kp |
| --- | --- | --- | --- | --- | --- | --- |
| *Cr* | 0.003 | 0.025 | 0.000075 | 0.5 | 500 | 0.002 |
| Fe | 0.7 | 0.2 | 0.14 |  |  | 0.001 |
| Mn | 0.024 | 0.04 | 0.00096 |  |  | 0.001 |
| Pb | 0.0014 | 0.3 | 0.00042 | 0.5 | 500 | 0.0001 |

Table S2. The results of HQ & HI Oral in each sample for adult and children

| Sampling | HQ_Fe | | HQ_Mn | | HQ_Cr | | HQ_Pb | | HI | |
| --- | --- | --- | --- | --- | --- | --- | --- | --- | --- | --- |
|  | Adults | Children | Adults | Children | Adults | Children | Adults | Children | Adults | Children |
| S1 | 2.58E-03 | 9.86E-03 | 2.74E-01 | 1.05E+00 | 1.31E-01 | 4.99E-01 | 2.15E-02 | 8.22E-02 | 4.28E-01 | 1.64E+00 |
| S2 | 7.84E-03 | 2.99E-02 | 2.37E-01 | 9.06E-01 | 1.91E-01 | 7.29E-01 | 6.46E-02 | 2.47E-01 | 5.01E-01 | 1.91E+00 |
| S3 | 3.87E-03 | 1.48E-02 | 1.44E-01 | 5.51E-01 | 8.04E-02 | 3.07E-01 | 2.15E-02 | 8.22E-02 | 2.50E-01 | 9.55E-01 |
| S4 | 1.00E-01 | 3.83E-01 | 4.03E-01 | 1.54E+00 | 1.21E-01 | 4.60E-01 | 6.46E-02 | 2.47E-01 | 6.89E-01 | 2.63E+00 |
| S5 | 9.04E-03 | 3.45E-02 | 5.39E-01 | 2.06E+00 | 4.02E-02 | 1.53E-01 | 4.31E-02 | 1.64E-01 | 6.31E-01 | 2.41E+00 |
| S6 | 5.73E-03 | 2.19E-02 | 5.46E-01 | 2.09E+00 | 1.00E-02 | 3.84E-02 | 2.80E-01 | 1.07E+00 | 8.42E-01 | 3.21E+00 |
| S7 | 8.61E-05 | 3.29E-04 | 2.90E-01 | 1.11E+00 | 3.01E-02 | 1.15E-01 | 4.52E-01 | 1.73E+00 | 7.72E-01 | 2.95E+00 |
| S8 | 5.38E-04 | 2.05E-03 | 5.42E-01 | 2.07E+00 | 1.21E-01 | 4.60E-01 | 4.74E-01 | 1.81E+00 | 1.14E+00 | 4.34E+00 |
| S9 | 1.38E-02 | 5.28E-02 | 3.15E-01 | 1.20E+00 | 2.21E-01 | 8.44E-01 | 7.53E-01 | 2.88E+00 | 1.30E+00 | 4.98E+00 |
| S10 | 9.90E-03 | 3.78E-02 | 5.31E-01 | 2.03E+00 | 1.51E-01 | 5.75E-01 | 4.31E-02 | 1.64E-01 | 7.35E-01 | 2.81E+00 |
| S11 | 5.81E-03 | 2.22E-02 | 2.74E-01 | 1.05E+00 | 2.01E-02 | 7.67E-02 | 2.15E-02 | 8.22E-02 | 3.21E-01 | 1.23E+00 |
| S12 | 1.01E-03 | 3.85E-03 | 6.18E-01 | 2.36E+00 | 7.03E-02 | 2.68E-01 | 1.72E-01 | 6.58E-01 | 8.61E-01 | 3.29E+00 |
| S13 | 4.31E-04 | 1.64E-03 | 1.54E-01 | 5.90E-01 | 1.00E-02 | 3.84E-02 | 4.74E-01 | 1.81E+00 | 6.39E-01 | 2.44E+00 |
| S14 | 8.61E-04 | 3.29E-03 | 1.28E-01 | 4.89E-01 | 2.01E-02 | 7.67E-02 | 2.58E-01 | 9.86E-01 | 4.07E-01 | 1.56E+00 |
| S15 | 1.08E-03 | 4.11E-03 | 3.89E-02 | 1.49E-01 | 2.01E-02 | 7.67E-02 | 7.10E-01 | 2.71E+00 | 7.70E-01 | 2.94E+00 |
| S16 | 8.22E-03 | 3.14E-02 | 4.92E-01 | 1.88E+00 | 2.01E-02 | 7.67E-02 | 6.89E-01 | 2.63E+00 | 1.21E+00 | 4.62E+00 |
| S17 | 2.30E-02 | 8.79E-02 | 5.56E-01 | 2.12E+00 | 3.01E-02 | 1.15E-01 | 5.38E-01 | 2.05E+00 | 1.15E+00 | 4.38E+00 |
| S18 | 7.88E-03 | 3.01E-02 | 5.37E-01 | 2.05E+00 | 1.11E-01 | 4.22E-01 | 5.81E-01 | 2.22E+00 | 1.24E+00 | 4.72E+00 |
| S19 | 1.42E-03 | 5.42E-03 | 1.78E-01 | 6.81E-01 | 3.01E-02 | 1.15E-01 | 2.15E-02 | 8.22E-02 | 2.31E-01 | 8.84E-01 |
| S20 | 4.52E-03 | 1.73E-02 | 6.54E-01 | 2.50E+00 | 2.11E-01 | 8.05E-01 | 3.66E-01 | 1.40E+00 | 1.24E+00 | 4.72E+00 |
| S21 | 6.29E-03 | 2.40E-02 | 3.14E-02 | 1.20E-01 | 1.11E-01 | 4.22E-01 | 3.44E-01 | 1.32E+00 | 4.93E-01 | 1.88E+00 |
| S22 | 7.15E-03 | 2.73E-02 | 2.47E-01 | 9.45E-01 | 2.01E-02 | 7.67E-02 | 1.94E-01 | 7.40E-01 | 4.68E-01 | 1.79E+00 |
| S23 | 1.34E-02 | 5.13E-02 | 6.43E-01 | 2.45E+00 | 1.21E-01 | 4.60E-01 | 1.08E-01 | 4.11E-01 | 8.85E-01 | 3.38E+00 |
| S24 | 2.33E-02 | 8.91E-02 | 6.93E-01 | 2.65E+00 | 2.21E-01 | 8.44E-01 | 6.03E-01 | 2.30E+00 | 1.54E+00 | 5.88E+00 |
| S25 | 5.55E-03 | 2.12E-02 | 4.91E-01 | 1.87E+00 | 1.31E-01 | 4.99E-01 | 6.46E-02 | 2.47E-01 | 6.92E-01 | 2.64E+00 |
| S26 | 8.61E-03 | 3.29E-02 | 6.17E-01 | 2.35E+00 | 1.41E-01 | 5.37E-01 | 1.08E-01 | 4.11E-01 | 8.73E-01 | 3.33E+00 |
| S27 | 1.25E-02 | 4.78E-02 | 7.67E-01 | 2.93E+00 | 1.91E-01 | 7.29E-01 | 1.51E-01 | 5.75E-01 | 1.12E+00 | 4.28E+00 |
| S28 | 1.54E-02 | 5.87E-02 | 4.77E-02 | 1.82E-01 | 1.51E-01 | 5.75E-01 | 7.32E-01 | 2.79E+00 | 9.46E-01 | 3.61E+00 |
| S29 | 1.94E-02 | 7.41E-02 | 3.64E-02 | 1.39E-01 | 3.11E-01 | **1.19E+00** | 5.81E-01 | 2.22E+00 | 9.48E-01 | 3.62E+00 |
| S30 | 7.41E-03 | 2.83E-02 | 1.62E-01 | 6.18E-01 | 9.04E-02 | 3.45E-01 | 6.03E-01 | 2.30E+00 | 8.63E-01 | 3.29E+00 |
| S31 | 2.20E-03 | 8.38E-03 | 3.77E-03 | 1.44E-02 | 1.00E-02 | 3.84E-02 | 7.32E-01 | 2.79E+00 | 7.48E-01 | 2.86E+00 |
| S32 | 2.67E-03 | 1.02E-02 | 2.26E-01 | 8.63E-01 | 2.01E-02 | 7.67E-02 | 7.53E-01 | 2.88E+00 | 1.00E+00 | 3.83E+00 |
| S33 | 5.38E-03 | 2.05E-02 | 2.67E-01 | 1.02E+00 | 1.21E-01 | 4.60E-01 | 7.75E-01 | 2.96E+00 | 1.17E+00 | 4.46E+00 |
| S34 | 3.92E-03 | 1.50E-02 | 1.22E-01 | 4.65E-01 | 1.71E-01 | 6.52E-01 | 8.18E-01 | 3.12E+00 | 1.11E+00 | 4.26E+00 |
| S35 | 5.64E-03 | 2.15E-02 | 3.92E-01 | 1.50E+00 | 2.21E-01 | 8.44E-01 | 2.37E-01 | 9.04E-01 | 8.55E-01 | 3.27E+00 |
| S36 | 4.31E-05 | 1.64E-04 | 7.03E-02 | 2.68E-01 | 1.91E-01 | 7.29E-01 | 4.52E-01 | 1.73E+00 | 7.13E-01 | 2.72E+00 |
| S37 | 5.94E-03 | 2.27E-02 | 2.37E-01 | 9.06E-01 | 7.03E-02 | 2.68E-01 | 6.46E-02 | 2.47E-01 | 3.78E-01 | 1.44E+00 |
| S38 | 1.34E-01 | 5.12E-01 | 3.23E-01 | 1.23E+00 | 2.21E-01 | 8.44E-01 | 7.96E-01 | 3.04E+00 | 1.47E+00 | 5.63E+00 |
| S39 | 1.18E-01 | 4.49E-01 | 2.86E-01 | 1.09E+00 | 5.02E-02 | 1.92E-01 | 7.53E-01 | 2.88E+00 | 1.21E+00 | 4.61E+00 |
| S40 | 9.09E-02 | 3.47E-01 | 7.92E-01 | 3.03E+00 | 2.11E-01 | 8.05E-01 | 1.51E-01 | 5.75E-01 | 1.24E+00 | 4.75E+00 |
| S41 | 1.29E-01 | 4.92E-01 | 1.59E-01 | 6.09E-01 | 2.21E-01 | 8.44E-01 | 4.95E-01 | 1.89E+00 | 1.00E+00 | 3.83E+00 |
| S42 | 3.14E-02 | 1.20E-01 | 1.41E-01 | 5.37E-01 | 1.91E-01 | 7.29E-01 | 7.75E-01 | 2.96E+00 | 1.14E+00 | 4.34E+00 |
| S43 | 1.53E-01 | 5.83E-01 | 9.92E-02 | 3.79E-01 | 1.41E-01 | 5.37E-01 | 1.08E-01 | 4.11E-01 | 5.00E-01 | 1.91E+00 |
| S44 | 1.56E-01 | 5.94E-01 | 3.55E-01 | 1.36E+00 | 2.21E-01 | 8.44E-01 | 7.10E-01 | 2.71E+00 | 1.44E+00 | 5.51E+00 |
| S45 | 1.23E-01 | 4.71E-01 | 1.63E-01 | 6.23E-01 | 2.21E-01 | 8.44E-01 | 8.40E-01 | 3.21E+00 | 1.35E+00 | 5.14E+00 |

Table S3. The results of HQ & HI Oral in each sample for adult and children

| Sampling | HQ_Fe | | HQ_Mn | | HQ_Cr | | HQ_Pb | | HI | |
| --- | --- | --- | --- | --- | --- | --- | --- | --- | --- | --- |
|  | Adults | Children | Adults | Children | Adults | Children | Adults | Children | Adults | Children |
| S1 | 3.04E-09 | 2.44E-08 | 1.61E-06 | 1.29E-05 | 2.46E-06 | 1.98E-05 | 1.69E-09 | 1.36E-08 | 4.07E-06 | 3.27E-05 |
| S2 | 9.21E-09 | 7.41E-08 | 1.39E-06 | 1.12E-05 | 3.59E-06 | 2.89E-05 | 5.06E-09 | 4.07E-08 | 5.00E-06 | 4.02E-05 |
| S3 | 4.55E-09 | 3.66E-08 | 8.48E-07 | 6.83E-06 | 1.51E-06 | 1.22E-05 | 1.69E-09 | 1.36E-08 | 2.37E-06 | 1.90E-05 |
| S4 | 1.18E-07 | 9.49E-07 | 2.37E-06 | 1.91E-05 | 2.27E-06 | 1.82E-05 | 5.06E-09 | 4.07E-08 | 4.76E-06 | 3.83E-05 |
| S5 | 1.06E-08 | 8.55E-08 | 3.17E-06 | 2.55E-05 | 7.55E-07 | 6.08E-06 | 3.37E-09 | 2.71E-08 | 3.93E-06 | 3.17E-05 |
| S6 | 6.73E-09 | 5.41E-08 | 3.21E-06 | 2.58E-05 | 1.89E-07 | 1.52E-06 | 2.19E-08 | 1.76E-07 | 3.43E-06 | 2.76E-05 |
| S7 | 1.01E-10 | 8.14E-10 | 1.70E-06 | 1.37E-05 | 5.67E-07 | 4.56E-06 | 3.54E-08 | 2.85E-07 | 2.31E-06 | 1.86E-05 |
| S8 | 6.32E-10 | 5.09E-09 | 3.19E-06 | 2.56E-05 | 2.27E-06 | 1.82E-05 | 3.71E-08 | 2.98E-07 | 5.49E-06 | 4.42E-05 |
| S9 | 1.62E-08 | 1.31E-07 | 1.85E-06 | 1.49E-05 | 4.16E-06 | 3.34E-05 | 5.90E-08 | 4.75E-07 | 6.08E-06 | 4.89E-05 |
| S10 | 1.16E-08 | 9.36E-08 | 3.12E-06 | 2.51E-05 | 2.83E-06 | 2.28E-05 | 3.37E-09 | 2.71E-08 | 5.97E-06 | 4.80E-05 |
| S11 | 6.83E-09 | 5.50E-08 | 1.61E-06 | 1.29E-05 | 3.78E-07 | 3.04E-06 | 1.69E-09 | 1.36E-08 | 1.99E-06 | 1.60E-05 |
| S12 | 1.18E-09 | 9.52E-09 | 3.63E-06 | 2.92E-05 | 1.32E-06 | 1.06E-05 | 1.35E-08 | 1.09E-07 | 4.97E-06 | 4.00E-05 |
| S13 | 5.06E-10 | 4.07E-09 | 9.07E-07 | 7.30E-06 | 1.89E-07 | 1.52E-06 | 3.71E-08 | 2.98E-07 | 1.13E-06 | 9.12E-06 |
| S14 | 1.01E-09 | 8.14E-09 | 7.53E-07 | 6.05E-06 | 3.78E-07 | 3.04E-06 | 2.02E-08 | 1.63E-07 | 1.15E-06 | 9.27E-06 |
| S15 | 1.26E-09 | 1.02E-08 | 2.29E-07 | 1.84E-06 | 3.78E-07 | 3.04E-06 | 5.56E-08 | 4.48E-07 | 6.63E-07 | 5.34E-06 |
| S16 | 9.66E-09 | 7.77E-08 | 2.89E-06 | 2.33E-05 | 3.78E-07 | 3.04E-06 | 5.40E-08 | 4.34E-07 | 3.33E-06 | 2.68E-05 |
| S17 | 2.71E-08 | 2.18E-07 | 3.27E-06 | 2.63E-05 | 5.67E-07 | 4.56E-06 | 4.22E-08 | 3.39E-07 | 3.90E-06 | 3.14E-05 |
| S18 | 9.26E-09 | 7.45E-08 | 3.16E-06 | 2.54E-05 | 2.08E-06 | 1.67E-05 | 4.55E-08 | 3.66E-07 | 5.29E-06 | 4.26E-05 |
| S19 | 1.67E-09 | 1.34E-08 | 1.05E-06 | 8.43E-06 | 5.67E-07 | 4.56E-06 | 1.69E-09 | 1.36E-08 | 1.62E-06 | 1.30E-05 |
| S20 | 5.31E-09 | 4.27E-08 | 3.84E-06 | 3.09E-05 | 3.97E-06 | 3.19E-05 | 2.87E-08 | 2.31E-07 | 7.84E-06 | 6.31E-05 |
| S21 | 7.39E-09 | 5.94E-08 | 1.84E-07 | 1.48E-06 | 2.08E-06 | 1.67E-05 | 2.70E-08 | 2.17E-07 | 2.30E-06 | 1.85E-05 |
| S22 | 8.40E-09 | 6.76E-08 | 1.45E-06 | 1.17E-05 | 3.78E-07 | 3.04E-06 | 1.52E-08 | 1.22E-07 | 1.85E-06 | 1.49E-05 |
| S23 | 1.58E-08 | 1.27E-07 | 3.78E-06 | 3.04E-05 | 2.27E-06 | 1.82E-05 | 8.43E-09 | 6.78E-08 | 6.07E-06 | 4.88E-05 |
| S24 | 2.74E-08 | 2.21E-07 | 4.07E-06 | 3.28E-05 | 4.16E-06 | 3.34E-05 | 4.72E-08 | 3.80E-07 | 8.30E-06 | 6.68E-05 |
| S25 | 6.53E-09 | 5.25E-08 | 2.88E-06 | 2.32E-05 | 2.46E-06 | 1.98E-05 | 5.06E-09 | 4.07E-08 | 5.35E-06 | 4.31E-05 |
| S26 | 1.01E-08 | 8.14E-08 | 3.62E-06 | 2.91E-05 | 2.64E-06 | 2.13E-05 | 8.43E-09 | 6.78E-08 | 6.29E-06 | 5.06E-05 |
| S27 | 1.47E-08 | 1.18E-07 | 4.51E-06 | 3.63E-05 | 3.59E-06 | 2.89E-05 | 1.18E-08 | 9.50E-08 | 8.12E-06 | 6.54E-05 |
| S28 | 1.81E-08 | 1.45E-07 | 2.80E-07 | 2.26E-06 | 2.83E-06 | 2.28E-05 | 5.73E-08 | 4.61E-07 | 3.19E-06 | 2.57E-05 |
| S29 | 2.28E-08 | 1.84E-07 | 2.14E-07 | 1.72E-06 | 5.85E-06 | 4.71E-05 | 4.55E-08 | 3.66E-07 | 6.14E-06 | 4.94E-05 |
| S30 | 8.70E-09 | 7.00E-08 | 9.52E-07 | 7.66E-06 | 1.70E-06 | 1.37E-05 | 4.72E-08 | 3.80E-07 | 2.71E-06 | 2.18E-05 |
| S31 | 2.58E-09 | 2.08E-08 | 2.21E-08 | 1.78E-07 | 1.89E-07 | 1.52E-06 | 5.73E-08 | 4.61E-07 | 2.71E-07 | 2.18E-06 |
| S32 | 3.14E-09 | 2.52E-08 | 1.33E-06 | 1.07E-05 | 3.78E-07 | 3.04E-06 | 5.90E-08 | 4.75E-07 | 1.77E-06 | 1.42E-05 |
| S33 | 6.32E-09 | 5.09E-08 | 1.57E-06 | 1.26E-05 | 2.27E-06 | 1.82E-05 | 6.07E-08 | 4.88E-07 | 3.90E-06 | 3.14E-05 |
| S34 | 4.60E-09 | 3.70E-08 | 7.16E-07 | 5.76E-06 | 3.21E-06 | 2.58E-05 | 6.41E-08 | 5.16E-07 | 4.00E-06 | 3.21E-05 |
| S35 | 6.63E-09 | 5.33E-08 | 2.30E-06 | 1.85E-05 | 4.16E-06 | 3.34E-05 | 1.85E-08 | 1.49E-07 | 6.48E-06 | 5.22E-05 |
| S36 | 5.06E-11 | 4.07E-10 | 4.13E-07 | 3.32E-06 | 3.59E-06 | 2.89E-05 | 3.54E-08 | 2.85E-07 | 4.04E-06 | 3.25E-05 |
| S37 | 6.98E-09 | 5.62E-08 | 1.39E-06 | 1.12E-05 | 1.32E-06 | 1.06E-05 | 5.06E-09 | 4.07E-08 | 2.73E-06 | 2.20E-05 |
| S38 | 1.57E-07 | 1.27E-06 | 1.90E-06 | 1.53E-05 | 4.16E-06 | 3.34E-05 | 6.24E-08 | 5.02E-07 | 6.27E-06 | 5.05E-05 |
| S39 | 1.38E-07 | 1.11E-06 | 1.68E-06 | 1.35E-05 | 9.44E-07 | 7.60E-06 | 5.90E-08 | 4.75E-07 | 2.82E-06 | 2.27E-05 |
| S40 | 1.07E-07 | 8.60E-07 | 4.66E-06 | 3.75E-05 | 3.97E-06 | 3.19E-05 | 1.18E-08 | 9.50E-08 | 8.74E-06 | 7.03E-05 |
| S41 | 1.51E-07 | 1.22E-06 | 9.37E-07 | 7.54E-06 | 4.16E-06 | 3.34E-05 | 3.88E-08 | 3.12E-07 | 5.28E-06 | 4.25E-05 |
| S42 | 3.69E-08 | 2.97E-07 | 8.26E-07 | 6.65E-06 | 3.59E-06 | 2.89E-05 | 6.07E-08 | 4.88E-07 | 4.51E-06 | 3.63E-05 |
| S43 | 1.79E-07 | 1.44E-06 | 5.83E-07 | 4.69E-06 | 2.64E-06 | 2.13E-05 | 8.43E-09 | 6.78E-08 | 3.41E-06 | 2.75E-05 |
| S44 | 1.83E-07 | 1.47E-06 | 2.09E-06 | 1.68E-05 | 4.16E-06 | 3.34E-05 | 5.56E-08 | 4.48E-07 | 6.48E-06 | 5.21E-05 |
| S45 | 1.45E-07 | 1.17E-06 | 9.59E-07 | 7.72E-06 | 4.16E-06 | 3.34E-05 | 6.58E-08 | 5.29E-07 | 5.32E-06 | 4.28E-05 |
